# CACLENS: A Multitask Deep Learning System for Enzyme Discovery

**DOI:** 10.1002/advs.202518063

**Published:** 2025-12-08

**Authors:** Xilong Yi, Yingzhu Tan, Huikang Lin, Guoqing Zhang, Ye Tian, Aibo Wu

**Affiliations:** ^1^ Shanghai Institute of Nutrition and Health University of Chinese Academy of Sciences Chinese Academy of Sciences Shanghai 200031 China

**Keywords:** biodegradation, enzyme screening, multitask deep learning, synthetic biology

## Abstract

Deep learning greatly advances large‐scale predictions of enzymatic structure, function, and properties. However, existing deep learning models remain limited in high‐performance screen of functional enzymes, due to a lack of multimodal learning and multitask prediction capabilities. To address these challenges, CACLENS (Cross‐Attention & Contrastive Learning‐enabled Enzyme Selection) is introduced, a multitask deep learning framework incorporating Customized Gate Control, contrastive learning, and cross‐attention mechanisms. CACLENS demonstrates robust performance across three key functions–reaction type classification, EC number prediction, and reaction feasibility assessment with fewer computational resources. These three functions are seamlessly incorporated into the enzyme screening pipeline for efficient screening of desired enzymes in biosynthesis and biodegradation processes, thereby significantly expediting the discovery of industrial enzymes. Using CACLENS, 10 potential degrading enzymes against Zearalenone (ZEN) are predicted and expressed, and one of them achieves a degradation efficiency of over 90% for ZEN and its analogue α‐ZOL. In addition, a user‐friendly web server for CACLENS is established and is accessible at https://ai.caclens.com/ for researchers to discover catalytic elements.

## Graphical abstract

CACLENS, a multimodal and multi‐task deep learning framework integrating cross‐attention, contrastive learning, and customized gate control, enables reaction type classification, EC number prediction, and reaction feasibility assessment. CACLENS accelerates functional enzyme discovery and identifies efficient Zearalenone (ZEN)‐degrading enzymes. A public web server is available at https://ai.caclens.com/.

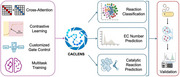

## 1 Introduction

Enzymes act as bio‐catalysts, driving and regulating nearly all chemical reactions and metabolic pathways within living organisms.^[^ ^1^, ^2^ ^]^ Enzymes with specific catalytic properties on substrates have been widely applied in various fields, such as food processing, natural products, pharmaceutical synthesis, and the degradation of environmental pollutants.^[^ ^3^ ^]^ Functional enzymes may exist in nature but need to be discovered through screening, while traditional experimental identification approaches are labor‐intensive and time‐consuming.^[^ ^4^ ^]^

By contrast, computational methods offer an alternative approach for enzyme screening. Machine learning (ML), especially deep learning (DL), has significantly advanced the prediction of enzyme structures and enzymatic properties, thereby speeding up functional enzyme screening processes. For instance, AlphaFold 3 leverages the Pairformer module as part of its new network architecture, enabling the prediction of enzyme‐substrate interactions and aiding in the identification of potential functional enzymes.^[^ ^5^ ^]^ Contrastive Learning Enabled Enzyme Annotation (CLEAN) can predict enzyme EC numbers more accurately than BLASTp and can screen enzymes with specific catalytic functions based on EC numbers.^[^ ^6^ ^]^ In addition, DL models such as ESP have been developed to predict enzyme‐substrate matching relationships for screening enzymes targeting specific substrates.^[^ ^7^ ^]^ Meanwhile, DL models have achieved the prediction of three key parameters in enzyme kinetics, *K_M_* ^[^ ^8^ ^]^ (Michaelis constant), *k_cat_* ^[^ ^9^ ^]^ (turnover number) and *k_cat_*/*K_M_* ^[^ ^10^ ^]^ (catalytic efficiency), which are crucial parameters for enzyme screening.

Although the aforementioned models demonstrate promising performance in certain scenarios, current DL models for enzyme screening still face challenges in directly generating a list of candidate enzymes capable of catalyzing a specific reaction. AlphaFold3 and other protein‐ligand interaction prediction tools cannot directly predict the feasibility of enzyme‐catalyzed reactions. Moreover, structure‐based DL models require high‐performance computing resources to process complex calculations, making large‐scale predictions computationally intensive. EC number prediction models also fall short in large‐scale predictions, as a single EC number may correspond to numerous distinct amino acid sequences. For example, as of March 2025, a UniProt^[^ ^11^ ^]^ search for enzymes with EC:3.1.1.1 yielded ≈8125 entries, but only 180 proteins were reviewed. Additionally, most prediction models of enzymes were designed for single‐task predictions, such as the prediction of enzyme‐substrate binding, *K_M_*, or *k_cat_*. The screening of functional enzymes typically requires the simultaneous prediction and integration of multiple aspects, including the feasibility of enzyme‐catalyzed reactions, enzyme function, and reaction type. However, no multitask model is currently available for comprehensive, high‐throughput enzyme screening.

Recent advancements in natural language processing (NLP) and large language models (LLMs) have enabled the efficient embedding of multilayered semantic structures of language.^[^ ^12^, ^13^ ^]^ Similarly, by treating amino acid sequences and the Simplified Molecular Input Line Entry System (SMILES) representations of molecules as “sentences”, the DL model can learn the semantic relationships either between proteins^[^ ^14^ ^]^ or between molecules. Pre‐trained LLMs in protein and chemical domains generate low‐dimensional embeddings that capture protein co‐evolutionary signals, structural details of small molecules, and other features, providing richer information than traditional encodings.^[^ ^15^, ^16^ ^]^ Meanwhile, the increasing availability of biological data, as well as the lower cost of genome and microbiome sequencing, has promoted the development of multimodal artificial intelligence to better address computational biology problems.^[^ ^17^, ^18^ ^]^ Currently, multimodal models have been used for tasks such as protein representation^[^ ^19^ ^]^ and function prediction.^[^ ^20^ ^]^ Therefore, the application of multimodal learning in protein screening helps overcome the limitations of single data sources, improving the enzyme screen performance through learning across different data types.

**Figure 1:**
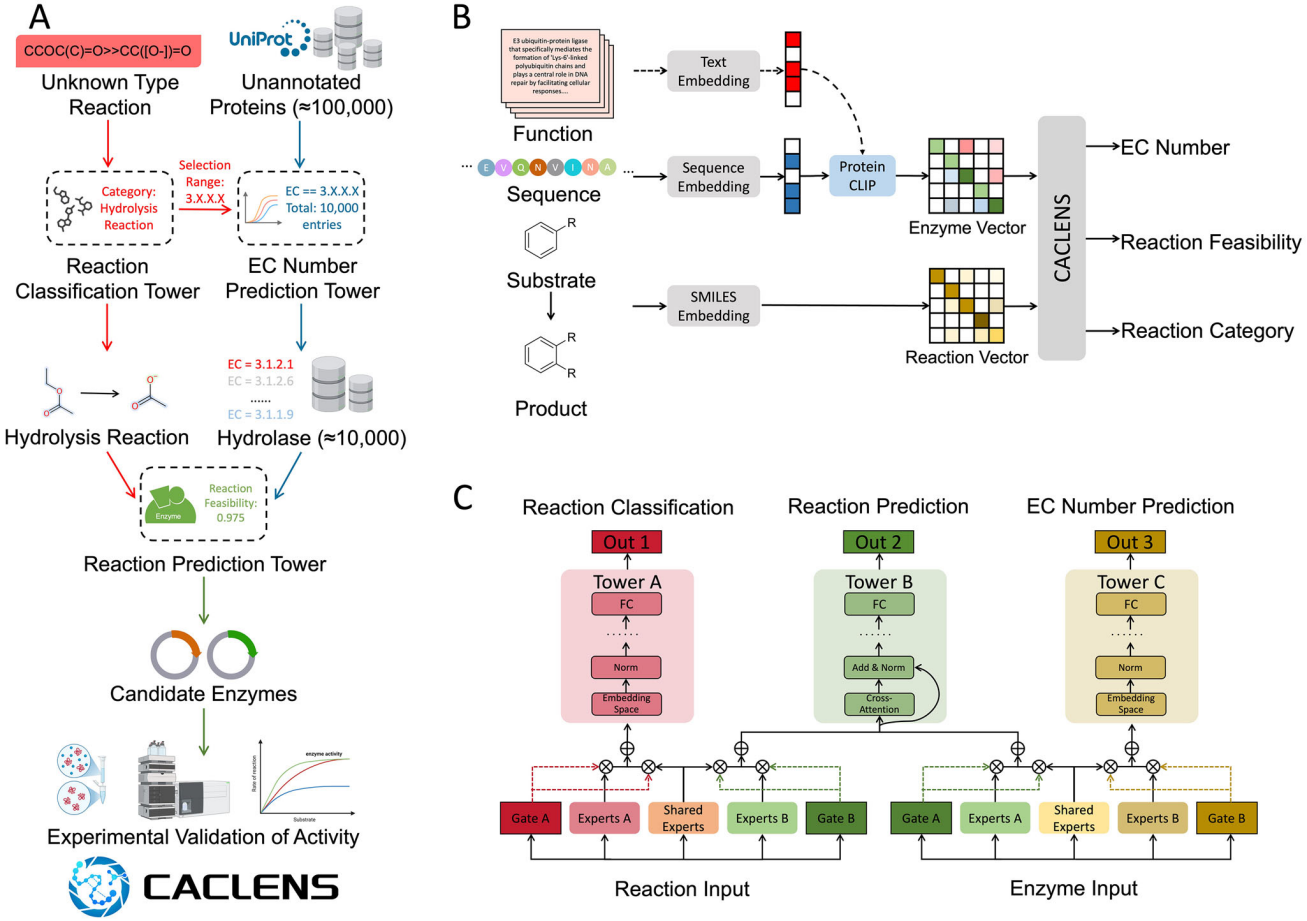
A framework for discovering novel functional enzymes based on a multitask model and prokaryotic protein expression. A) Workflow for discovering novel functional enzymes. Take the screening of hydrolytic enzymes as an example. B) Overview of the model input and output. After the protein sequences are encoded, the pre‐trained ProteinCLIP model will embed the protein's encoding into different spaces based on the enzyme function text corresponding to the protein sequences (indicated by the dashed arrow). Meanwhile, the encodings of the reactants and products are concatenated. C) Multitask framework of CACLENS. The “Embedding Space” represents the use of a contrastive learning strategy to optimize the distance relationship for learning spatial embeddings. “Add & Norm” refers to the normalization step applied during training, while “FC” refers to the fully connected layer.

To facilitate enzyme screening efficiency via a novel data‐driven strategy, we developed Cross‐Attention & Contrastive Learning‐enabled Enzyme Selection (CACLENS), a DL system built on a multi‐task learning (MTL) framework integrating Customized Gate Control (CGC),^[^ ^21^ ^]^ contrastive learning, and a cross‐attention mechanism. CACLENS stands apart from existing DL models by implementing multimodal learning through a multitask architecture, incorporating information such as biochemical reactions, protein sequences, and protein functions. This enables CACLENS to achieve more accurate chemical reaction representation while also predicting the enzyme's EC number and assessing the feasibility of the given protein catalyzing the specified reaction. These functionalities of CACLENS were integrated into the enzyme screening system, facilitating the identification of enzymes for a wide range of reactants and products (**Figure** **1**A). Additionally, a case study of screening mycotoxin Zearalenone (ZEN) degradation enzymes was performed. CACLENS was employed to assist in predicting potential degrading enzymes against ZEN. In all, CACLENS would boost functional enzyme discovery in various scenarios, such as metabolic pathway design for biosynthesis/biodegradation,^[^ ^22^ ^]^ environmental pollutant degradation,^[^ ^23^ ^]^ and industrial enzyme mining.^[^ ^24^ ^]^

## 2 Results

### 2.1 Development and Evaluation of CACLENS

The datasets for reaction feasibility prediction were collected from the UniProt^[^ ^11^ ^]^ and Rhea^[^ ^25^ ^]^ databases. After data cleaning, only reactants and products were retained from each reaction record (Figure S1, Supporting Information). In total, 164 837 unique protein sequences and 17 909 distinct chemical reactions were collected, yielding 948 868 positive samples. To address the issue of negative sample data,^[^ ^26^ ^]^ approximately ten times as many negative samples were generated (see Experimental Section for details). A total of 379 548 samples, consisting of 20% of the positive samples and an equal number of negative samples, were extracted as the test set.

CACLENS simultaneously trains three tasks–chemical reaction representation, EC number prediction, and reaction feasibility prediction–within a single model. As shown in Figure 1B, CACLENS accepts reactants, products, and candidate protein sequences as inputs. A function‐centric embedding for each protein sequence was produced from its functional annotations using the pre‐trained ProteinCLIP model, thus introducing multimodal protein representations. To avoid negative transfer and the seesaw phenomenon,^[^ ^27^, ^28^, ^29^ ^]^ a MTL architecture based on CGC was successfully applied to CACLENS (Figure 1C). Specifically, extracted features are processed through expert layers, gate control layers, and task‐specific tower layers to generate the final outputs. The Reaction Classification Tower and the EC Number Prediction Tower of CACLENS are optimized simultaneously using contrastive learning. The reaction representations output by the Reaction Classification Tower are used for the subsequent reaction classification task. Meanwhile, the Reaction Prediction Tower is optimized using a binary classification loss function with smoothed labels, primarily for reaction feasibility prediction.

To investigate the impact of different protein and molecular encoders on the performance of CACLENS, two pre‐trained DL embedding models were independently applied to protein sequences and small molecules. This results in four encoding configurations: ProtT5^[^ ^30^ ^]^ + UniMol,^[^ ^16^ ^]^ ProtT5 + MoLFormer,^[^ ^31^ ^]^ ESM‐2^[^ ^32^ ^]^ + UniMol, and ESM‐2 + MoLFormer. These four models differ only in the encoding of proteins and small molecules, while keeping the model architecture, initialization, and training procedure unchanged. They correspond to four variants: CACLENS‐TU, CACLENS‐TF, CACLENS‐EU, and CACLENS‐EF.

Based on the above work, a high‐throughput enzyme screening workflow is constructed and proceeds as follows (Figure 1A): First, arbitrary reactants and products (with unknown reaction types) along with unannotated enzymes are input into CACLENS. Next, the Reaction Classification Tower predicts the reaction type and infers the corresponding EC number range, which is then used by the EC Number Prediction Tower to filter candidate enzymes. Finally, the Reaction Prediction Tower assesses whether candidates can catalyze the target reaction, with their activity further validated by wet‐lab experiments.

### 2.2 Benchmarking CACLENS on Reaction Representation and Classification

Computational methods have been applied to the representation of chemical reactions. For example, Schneider et al.^[^ ^33^ ^]^ proposed a method based on difference fingerprints, while Ghiandoni et al. ^[^ ^34^ ^]^ employed reaction change graphs as feature representations. These methods can effectively encode reactions, and their encoding capability was validated through chemical reaction classification tasks.

However, these reaction encoding methods have shown limitations when applied to the feasibility analysis of enzyme‐catalyzed reactions, as enzymes can catalyze the same chemical transformation through entirely different mechanisms, a phenomenon known as convergent evolution.^[^ ^35^ ^]^ This makes encoding strategies that rely solely on substrate‐product differences difficult to generalize across diverse enzyme systems. Such limitations are particularly evident in reactions where substrate changes are minimal, yet enzyme specificity is high. Moreover, most existing reaction‐centric encoding approaches fail to incorporate enzyme information—limiting their ability to capture the enzyme's true catalytic role and constraining their applicability to enzyme screening tasks—while the direct encoding of common cofactors (e.g., ATP) in enzyme‐catalyzed reactions may further hinder a deep learning model's ability to learn the genuine characteristics of enzyme catalysis.^[^ ^36^, ^37^ ^]^

To better represent enzyme‐catalyzed reactions, all reaction data in CACLENS were subjected to a data‐cleaning process, and reactions were represented by encoding individual small molecules and then concatenating them (see Supplementary Methods for details). Specifically, by initially encoding small molecules with UniMol or MoLFormer and incorporating them into the reaction encoding, CACLENS learns a comprehensive representation of the reaction through the shared expert module of the CGC system, effectively capturing both the chemical transformation and the enzymatic context.

**Figure 2:**
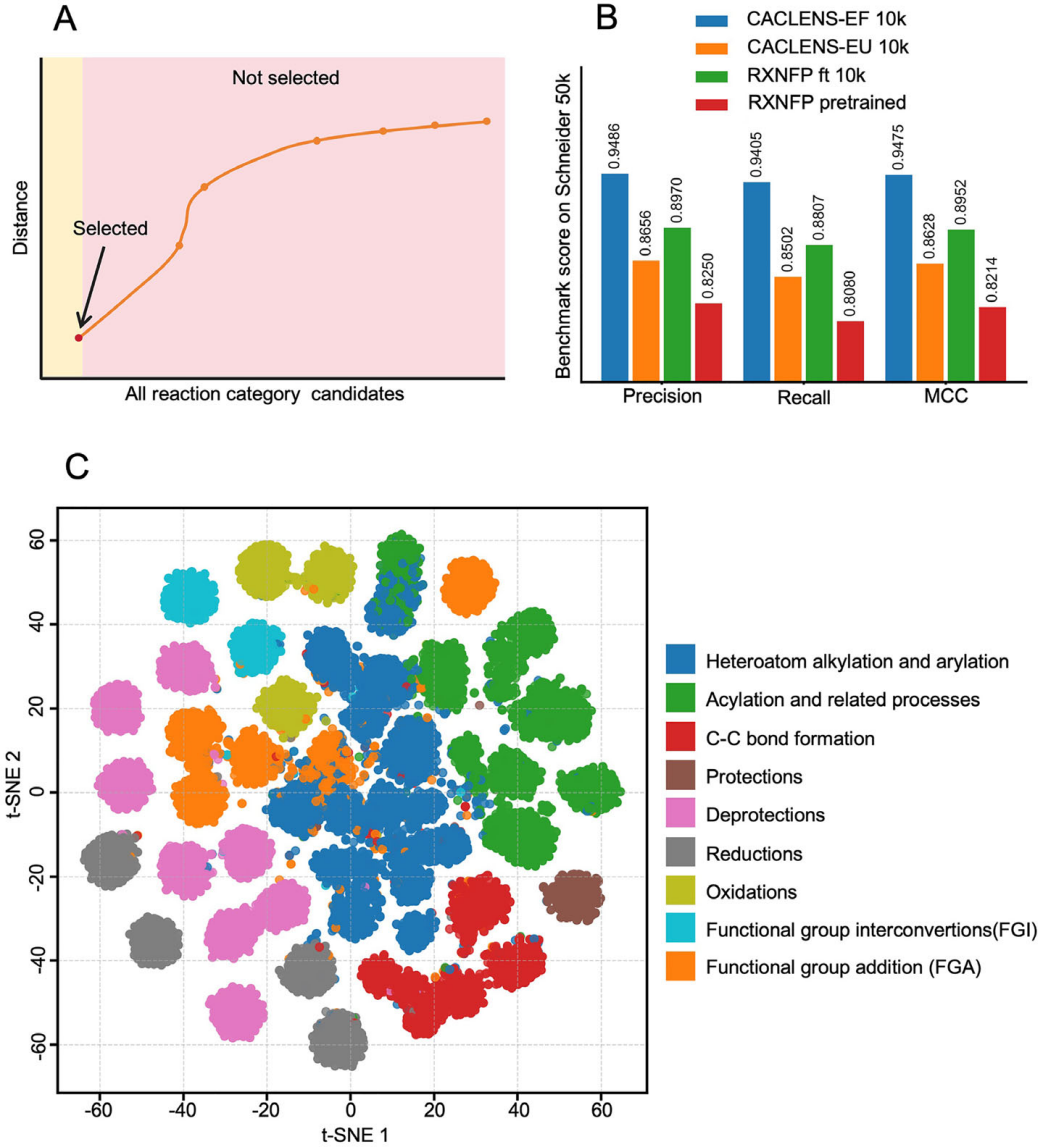
Benchmarking CACLENS on Reaction Representation and Classification. A) Euclidean‐distance‐based method for reaction type prediction. During the inference phase, the Reaction Classification Tower outputs the reaction fingerprint for a given reaction. The reaction type of the query is determined by calculating the Euclidean distance between the query reaction's fingerprint and the fingerprints of known candidate reaction types. B) Comparison of the precision, recall, and MCC performance between CACLENS and *rxnfp*. C) 2D visualization of the CACLENS‐EF reaction embeddings was generated using t‐SNE. Each dot in the plot represents a chemical reaction, and the color represents the reaction superclass.

To evaluate whether CACLENS's reaction encoding strategy is efficient and accurate for enzyme screening tasks, the approach of Schwaller et al. ^[^ ^38^ ^]^ was adopted, with ≈20% (10 000 reactions) extracted from the cleaned Schneider 50k dataset as the training set and the remaining portion used as the test set. During the training phase, contrastive learning is applied to the Reaction Prediction Tower, and both the EC Number Tower and Reaction Prediction Tower are optimized to influence reaction representations based on shared expert knowledge. During the inference phase, reaction classification is performed either using logistic regression^[^ ^38^ ^]^ or by computing the Euclidean distance (**Figure** **2**A).

As a result, for the Schneider 50k dataset, CACLENS‐EF (10k) achieved the highest classification performance using logistic regression, with a precision of 0.9486, recall of 0.9405, and Matthews Correlation Coefficient (MCC)^[^ ^39^, ^40^ ^]^ of 0.9475. In contrast, *rxnfp* (pretrained) had a precision of 0.8250, while fine‐tuning on the training set improved *rxnfp* (10k) to a precision of 0.8970. However, CACLENS‐EU (10k) performed poorly in reaction classification, with a precision of only 0.8656, a recall of 0.8502, and an MCC of 0.8628 (Figure 2B). Using the logistic regression results from the randomly shuffled reaction encoding output as a comparison (Table S1, Supporting Information), we thoroughly compared the performance of CACLENS‐E U (10k, Table S2, Supporting Information) and CACLENS‐E F (10k, Table S3, Supporting Information) across 50 chemical reaction categories. Among them, CACLENS‐EF (10k) achieved a precision above 0.9 in 40 reaction types, with the precision for Nitrile reduction reactions reaching 0.9925 and the recall reaching 1 (Table S3, Supporting Information). Based on the characteristics of contrastive learning, the Euclidean distance between the query reaction embedding and each embedding in the training set was computed, and the label of the nearest neighbor was assigned as the prediction (Figure 2A). The results based on Euclidean distance were comparable to those obtained with logistic regression (Tables S4 and S5, Supporting Information). However, the performance of *rxnfp* (pretrained) and *rxnfp* (10k) across the fifty reaction types was not ideal. Among them, *rxnfp* (pretrained) achieved a precision greater than 0.9 in 24 reaction types (Table S6, Supporting Information), while *rxnfp* (10k) achieved this in 31 types (Table S7, Supporting Information). The above reaction classification results are all presented through confusion matrix visualizations (Figures S2–S8, Supporting Information). For similar reactions, such as Bromo Suzuki coupling and Bromo Suzuki‐type coupling, *rxnfp* (10k) showed only moderate performance, with precisions of 0.409 and 0.5746 (Table S7, Figure S8, Supporting Information), respectively. In contrast, CACLENS‐EF achieved 0.8012 and 0.937 (Table S3, Figure S4, Supporting Information), representing a clear improvement. Nevertheless, the effective classification of such closely related reactions remains a challenge.

Figure 2C shows the 2D visualization of reaction embeddings of Schneider 50k generated by CACLENS‐EF using t‐Distributed Stochastic Neighbor Embedding (t‐SNE).^[^ ^41^ ^]^ Similarly, reaction embeddings were visualized using the TMAP algorithm^[^ ^42^ ^]^ and the Faerun visualization library.^[^ ^43^ ^]^ The t‐SNE and TMAP (Figures S9 and S10, Supporting Information) results show that CACLENS‐EF effectively groups reactions of similar types based on their embedding information.

### 2.3 CACLENS Performance Evaluation on EC Number Prediction

The ProteinCLIP^[^ ^44^ ^]^ model has already been implemented to enhance protein embedding DL models, such as ESM‐2 and ProtT5. It is interesting to integrate natural language descriptions of enzyme functions, protein sequences, and chemical reaction information, using the shared expert module of the CGC system and ProteinCLIP, to achieve a more comprehensive protein representation.

CACLENS's encoding ability for protein function was tested on EC number prediction, a multi‐label task. Five benchmark datasets constructed by Yu et al.^[^ ^6^ ^]^–split10, split30, split50, split70, and split100–are utilized in the EC number prediction task of CACLENS. These datasets were constructed based on the similarity threshold of different sequences. For instance, in the split50 dataset, each sequence in the test set shares no more than 50% similarity with any sequence in the training set after an 80/20 train‐test split. During the training of the EC number prediction task, the test set partitioning and training procedure of CLEAN^[^ ^6^ ^]^ were strictly followed.

**Figure 3:**
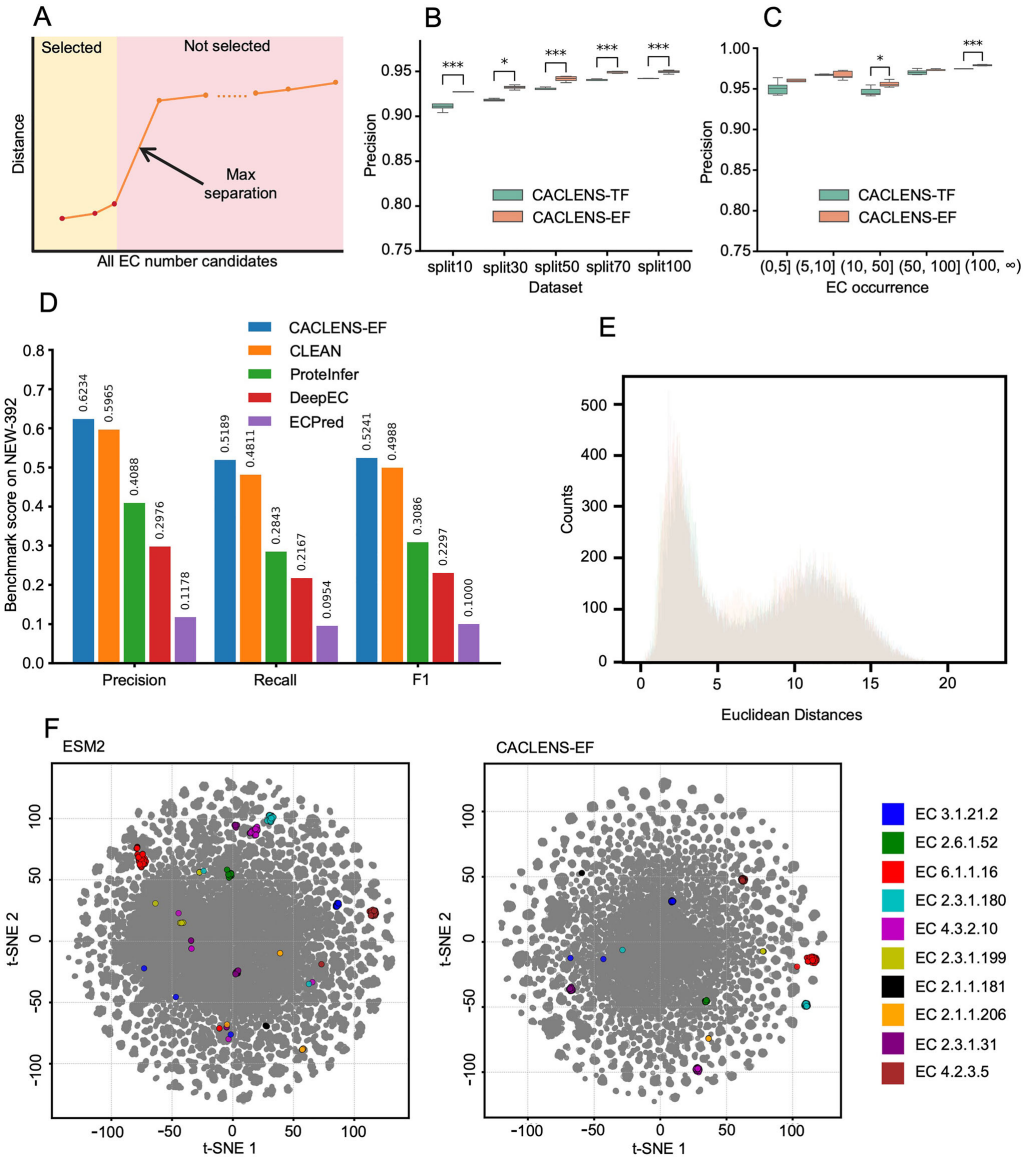
CACLENS Performance Evaluation on EC Number Prediction. A) EC number prediction method. According to the Max‐Separation selection algorithm, an enzyme can be predicted to have one or more EC numbers. B) The five‐fold cross‐validation results of the precision for CACLENS‐TF and CACLENS‐EF on five datasets. C) The precision binned plot of five‐fold cross‐validation for CACLENS‐TF and CACLENS‐EF (on the split70 dataset). D) A comparison of the performance of CACLENS‐EF with CLEAN, ProteInfer, DeepEC, and ECPred using the New‐392 dataset. E) The histogram of the distance distribution. The *x*‐axis represents the Euclidean distance, and the *y*‐axis represents the enzyme count. F) Comparison of the embeddings from the EC Number Prediction Tower and ESM‐2. A 2D visualization of the split70 dataset embeddings using t‐SNE. Each dot in the plot represents a single enzyme. Ten enzymes were randomly selected and highlighted, with each color representing a different EC number, while the remaining enzymes are shown in gray.

Unlike the single‐label reaction classification, we used the Max‐Separation^[^ ^6^ ^]^ method during inference for EC number prediction (**Figure** **3**A). The results showed that, in all cases, the precision of the CACLENS‐EF was higher than that of the CACLENS‐TF (Figure 3B). In the split100 dataset, the CACLENS‐EF achieved an average precision of 0.9495, while the CACLENS‐TF had an average precision of 0.9415 (Figure 3B). Meanwhile, the CACLENS‐EF achieved higher recall (Figure S11A, Supporting Information) and F1 score (Figure S11B, Supporting Information) than the CACLENS‐TF. During the five‐fold cross‐validation process, all proteins in the test set were divided into five subsets based on the occurrence of their EC numbers in the training set: (0, 5], (5, 10], (10, 50], (50, 100], and (100, ∞). For example, the subset (0, 5] represents the portion of the test set where the EC number appears more than 0 times but no more than 5 times in the training set. In the split70 dataset, both the CACLENS‐EF and the CACLENS‐TF models achieved high precision (Figure 3C) and ROC‐AUC (Figure S11C, Supporting Information) performance across all subsets based on the occurrence. Notably, both models achieved relatively high ROC‐AUC (CACLENS‐EF with an average of 0.9777, and CACLENS‐TF with an average of 0.9738) even in the subset (0, 5]. Even with the sequence similarity thresholds in the test set reduced to 50%, as in the split50 dataset, both models still demonstrated outstanding precision (Figure S11D, Supporting Information) and ROC‐AUC (Figure S11E, Supporting Information) performance.

CACLENS‐EF, which performed better across the five datasets (split10–100), was selected and compared with four state‐of‐the‐art EC number annotation tools: CLEAN, ProteInfer,^[^ ^45^ ^]^ DeepEC,^[^ ^46^ ^]^ and ECPred.^[^ ^47^ ^]^ Following Yu et al.,^[^ ^6^ ^]^ the New‐392 dataset and the New‐392+Price‐149 dataset were employed to evaluate EC number prediction on completely unknown proteins. The comparison of these models was also conducted in strict accordance with CLEAN.

For the New‐392 dataset, the CACLENS‐EF achieved the highest performance across various multilabel precision metrics, with a precision of 0.6234, a recall of 0.5189, and an F1 score of 0.5241 (Figure 3D). Compared to the CLEAN, the precision, recall, and F1 score improved by 4.5%, 7.9%, and 5.1%, respectively. In contrast, ProteInfer and DeepEC performed worse (Figure 3D). For the New‐392+Price‐149 dataset, the CACLENS‐EF still achieved the best performance (Figure S12, Supporting Information). Furthermore, based on the occurrence of EC numbers in the New‐392+Price‐149 dataset, it was divided into five subsets: (0, 5], (5, 10], (10, 50], (50, 100], and (100, ∞). For proteins with an EC number occurrence of less than 10, the CACLENS‐EF achieved higher precision (Figure S13A, Supporting Information) and recall (Figure S13B, Supporting Information) compared to CLEAN. For proteins with an EC number occurrence greater than 50, the precision and recall of the CACLENS‐EF model were close to those of CLEAN (Figure S13A,B, Supporting Information). This indicated that CACLENS‐EF has a greater advantage in predicting EC numbers for proteins that are rarely reported.

At the same time, to quantify the confidence of the prediction results, a two‐component Gaussian mixture model (GMM)^[^ ^6^ ^]^ was fitted to the distribution of Euclidean distances between enzyme embeddings in the split100 dataset, generated by the EC number prediction tower of CACLENS‐EF. The results are shown in Figure 3E. The left peak is formed by enzymes with the same EC number, while the right peak is formed by enzymes with different EC numbers.

To better visualize the protein embeddings learned by CACLENS‐EF, t‐SNE was also applied to reduce the high‐dimensional embeddings of the split100 dataset from CACLENS‐EF to a 2D space. Compared to the embeddings generated by ESM‐2 before contrastive learning, the visualization of CACLENS‐EF exhibited more distinct clusters (Figure 3F).

### 2.4 Assessing CACLENS Performance on Reaction Feasibility Prediction

To benchmark CACLENS against other existing models for reaction feasibility prediction, ESP^[^ ^7^ ^]^ and EnzRank,^[^ ^48^ ^]^ which represent state‐of‐the‐art models, were selected as baseline models.

**Figure 4:**
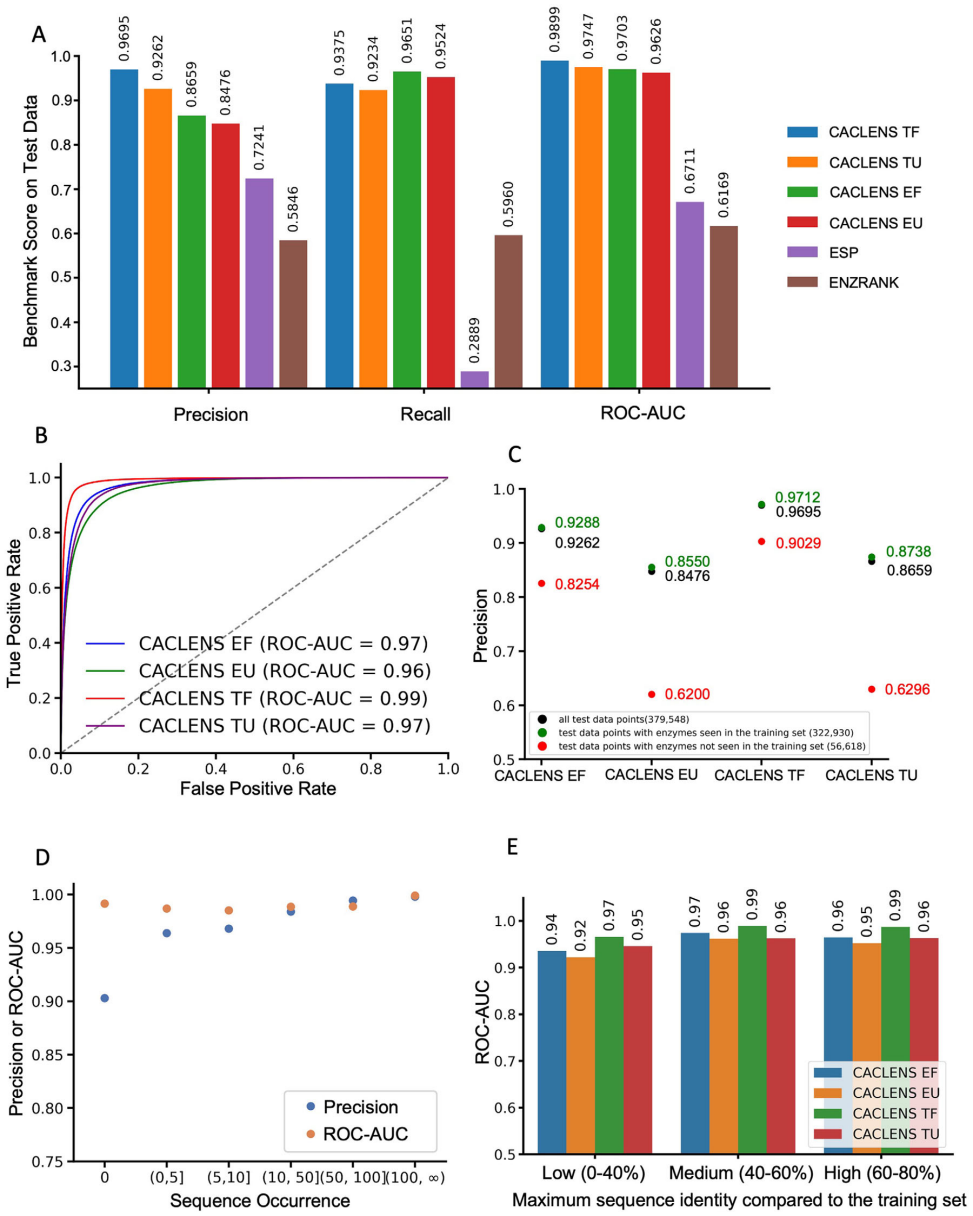
Assessing CACLENS Performance on Reaction Feasibility Prediction. A) The performance in terms of precision, recall, and ROC‐AUC parameters, comparing CACLENS (CACLENS‐TF, TU, EF, EU) with ESP and EnzRank on the entire test set of 379 548 samples. B) The ROC curves of CACLENS, with the dashed line representing the ROC curve of a model that simulates random classification. C) The performance of CACLENS in terms of precision on the test set. The test set is divided into two parts based on whether the enzymes appear in the training set. D) The precision and ROC‐AUC binned plot for CACLENS‐TF, with bins organized according to the frequency of sequence appearances in the training set. E) ROC‐AUC performance of CACLENS on subsets divided according to the maximum sequence identity between the test set and the training set.

As shown in **Figures** **4**A and S14A (Supporting Information), CACLENS (CACLENS‐TU, CACLENS‐TF, CACLENS‐EU, and CACLENS‐EF) achieved the highest levels across multiple parameters. Among them, CACLENS‐TF achieved the most robust performance, with a precision of 0.9695, a recall of 0.9375, and a ROC‐AUC of 0.9899 on the test set of 379 548 samples. (Figure 4A). Moreover, the PRC‐AUC, F1 score, and MCC of CACLENS‐TF were 0.9886, 0.9532, and 0.9085, respectively, which were the highest among all models (Figure S14A, Supporting Information). In contrast, ESP and EnzRank performed poorly. ESP achieved a precision of 0.7241, a recall of 0.2889, and an MCC of 0.2197. EnzRank had a precision of 0.5846, a recall of 0.5960, and an MCC of 0.1689. To visually compare the differences among the four CACLENS models, their ROC curves were plotted. Consequently, CACLENS‐TF achieved the highest True Positive Rate (TPR) at the lowest False Positive Rate (FPR), demonstrating the best performance (Figure 4B).

Since it is common for a single enzyme to catalyze multiple reactions, the test set may contain some enzyme sequences that also appear in the training set. To evaluate whether CACLENS can accurately predict the catalytic reaction feasibility of new enzymes, the test set was divided into two subsets based on whether the enzyme sequence appeared in the training set. The precision (Figure 4C), recall (Figure S14B, Supporting Information), and F1 score (Figure S14C, Supporting Information) of CACLENS were evaluated separately on the two subsets. As a result, CACLENS‐TF achieved the highest parameter performance in both subsets. Even for new enzymes (a total of 56 618), CACLENS‐TF still attained a precision of 0.9029, a recall of 0.8881, and an F1 score of 0.8954. The other three CACLENS exhibited a significant decline in parameter performance for sequences do not present in the training set. Among them, CACLENS‐EU's precision dropped to 0.6200 (Figure 4C), recall decreased to 0.8717 (Figure S14B, Supporting Information), and F1 score declined to 0.7246 (Figure S14C, Supporting Information). Furthermore, based on the performance of the six subsets of the test set, which were divided according to the frequency of sequence appearances in the training set, CACLENS also demonstrated stable ROC‐AUC and precision performance (Figure 4D).

To further evaluate the generalization ability of CACLENS and to avoid overestimating its performance, all test sequences were compared with the training set for identity. Test data with sequence identity below 80% were then stratified into three subsets: low identity (0–40%), medium identity (40–60%), and high identity (60–80%). Although low‐identity sequences led to a decline in CACLENS performance, CACLENS still maintained high ROC‐AUC values on the distantly related enzymes (with sequence identity below 40%) (Figure 4E), with CACLENS‐TF reaching 0.97.

### 2.5 High‐Throughput Prediction of Functional Enzymes and Experimental Validation

**Figure 5:**
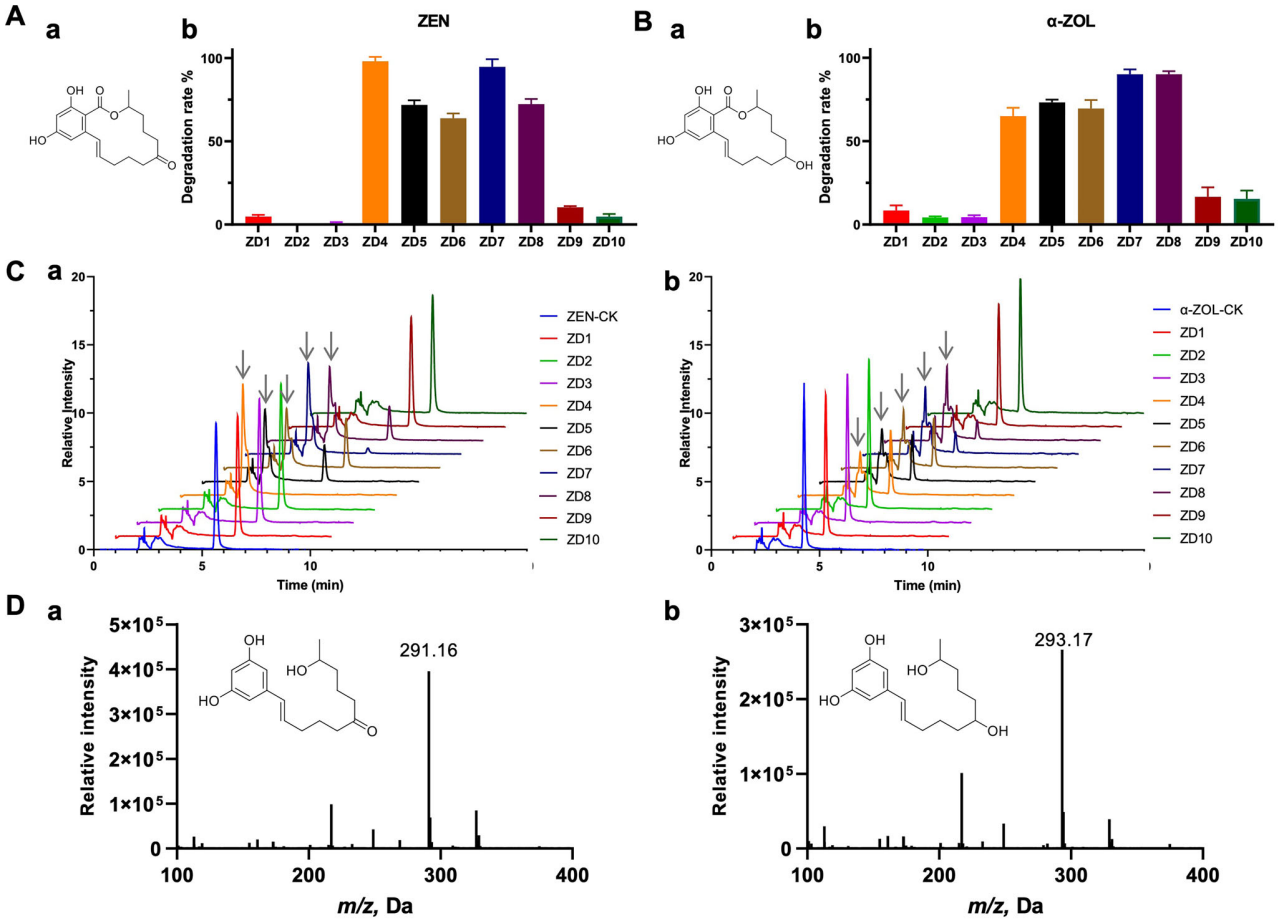
Experimental verification of candidate ZEN degradation enzymes. A) Chemical structure of ZEN a) and ZEN degradation assay b). B) Chemical structure of α‐ZOL (a) and α‐ZOL degradation assay (b). C) HPLC profile of ZEN and its degradation products(a), α‐ZOL and its degradation products (b). D) HRMS analysis of ZEN degradation product (a) and α‐ZOL degradation product (b). Candidates ZD4–ZD8, whose degradation rates exceeded 50% on ZEN and α‐ZOL, were further analyzed by One‐way ANOVA analysis followed by Tukey's Test (α < 0.05). Bars with different letters were significantly different.

To test the screening ability of CACLENS for functional enzymes, we used ZEN as an example to predict and screen for degrading enzymes. ZEN (structure shown in **Figure** **5**A,a) is a mycotoxin produced by *Fusarium* fungi, posing a serious threat to the health of humans and livestock.^[^ ^49^ ^]^ Biological degradation with strong targeting and safety properties has gained extensive attention,^[^ ^50^ ^]^ but its application is limited by issues such as catalytic efficiency. In recent years, the known ZEN‐degrading enzymes and microbes were majorly screened from nature, while the development of DL has offered possibilities for mining novel and highly efficient ZEN‐degrading enzymes.

The three functions of CACLENS trained simultaneously were all used for enzyme screening, as detailed in the Experimental Section. According to the prediction of CACLENS, 10 potential ZEN degradation enzymes (ZD 1–10) were selected for the next experiments (Table S8, Supporting Information). The candidate genes were synthesized and then expressed with *E. coli* BL21 system. The enzymes were purified and reacted with mycotoxin ZEN in Tris‐HCl buffer. Overall, 50% of the predicted enzymes exhibited catalytic activities on ZEN, of which ZD4 and ZD7 eliminated >90% of ZEN (Figure 5A,b). To further confirm if the candidates could degrade ZEN's major reduced form α‐ZOL (structure shown in Figure 5B,a), we treated α‐ZOL with purified enzymes, and similar trends were obtained. Five enzymes, ZD4‐ZD8 could catalyze >50% of α‐ZOL, while ZD7 and ZD8 eliminated >90% of α‐ZOL (Figure 5B,b). A new peak with retention time of 3.0 min appeared in the HPLC profile of ZEN treated samples (Figure 5C,a), while for α‐ZOL, the new peak occurred in 2.8 min (Figure 5C,b), which were the expected degradation products and ZEN and α‐ZOL respectively. High‐resolution mass spectrometry (HRMS) then was used to analyze these degradation metabolites. The ZEN metabolite with an *m/z* [M‐H]‐ 291.16 was detected in samples of ZD4‐ZD8 (Figure 5D,a), while the product of α‐ZOL with an *m/z* [M‐H]‐ 293.17 was detected in samples of ZD4‐ZD8 (Figure 5D,b). Overall, ZD1‐3 and ZD9‐10 exhibit extremely low degradation rates on both ZEN and α‐ZOL, while ZD7 possesses higher catalytic activity on these mycotoxins. Next, we tested the degradation effect of ZD7 on mycotoxins (ZEN and α‐ZOL) contaminated wheat flour. Though real food matrix may influence the enzymatic degradation activity, ZD7 still degraded >80% of ZEN and α‐ZOL in wheat flour (Figure S15, Supporting Information), indicating its great potential to eliminate ZEN family mycotoxins in contaminated foodstuffs. These results indicate the feasibility of CACLENs to accelerate screen novel enzymes to degrade mycotoxin ZEN, as well as its reduced form α‐ZOL.

## 3 Discussion

Traditional experiment‐based enzyme screening methods are time‐consuming and labor‐intensive. Deep learning (DL) offers new insights for enzyme screening, including models like AlphaFold3 and CLEAN for accurate enzyme structure and function prediction, and the ESP model for predicting enzyme‐substrate interactions. These models have demonstrated good performance in certain scenarios, but DL models for enzyme screening still face challenges such as high computational resource demands, inability to accurately predict enzyme functions, and limited learning of enzyme‐catalyzed reactions. Meanwhile, computational methods have been applied to the representation of chemical reactions, but traditional reaction encoding methods have limitations in capturing the true catalytic role of enzymes in enzyme‐catalyzed reactions. Similarly, DL models for enzyme encoding, such as ESM‐2, lack information on protein function and protein‐catalyzed reactions.

In this study, we developed a multi‐task enzyme screening system, CACLENS, based on CGC system using publicly available databases Rhea and UniProt, the EC number dataset (split10, split30, split50, split70, and split100), and the reaction classification dataset (Schneider 50k). While using DL models to encode proteins and small molecules, we leveraged shared experts of the CGC system and ProteinCLIP model to enable CACLENS to simultaneously learn information on protein sequences, natural language of protein functions, chemical reactions, and enzyme‐reaction interactions. Based on this architecture, reaction classification, EC number prediction, and reaction feasibility assessment were simultaneously performed within an independent model, enabling both high through and accurate enzyme screening.

In the reaction classification performance comparison, since CACLENS is more focused on substrate‐to‐product conversions catalyzed by enzymes, while molecules like ADP, ATP, and H⁺ are not considered primary reactants but common cofactors, all reaction inputs were subjected to a cleaning procedure. Moreover, no models are currently dedicated to the classification and encoding of enzyme‐catalyzed reactions. Therefore, in the main application scenario of biocatalytic reaction encoding (i.e., data cleaning by removing cofactors), the Schneider 50k dataset was partitioned following the same scheme as *rxnfp* (10k), with 10 000 reactions used for training and 40 000 for testing. Through comparison with *rxnfp* (10k) and *rxnfp* (pretrained), the results indicate that the encoding strategy of CACLENS is more suitable for reaction representation, classification, subsequent feasibility prediction, and for matching appropriate enzymes to completely unknown reactions (Figure 1A) after data cleaning by removing cofactors. Therefore, the comparison with *rxnfp* highlights the applicability in biocatalytic scenarios rather than general ones, and thus the accuracy reported in the original *rxnfp* paper was not cited. In future work, we will explore the feasibility of extending the reaction encoding and classification capabilities of CACLENS to broader, more general applications. In the comparison of EC number prediction performance, all datasets, training, and model evaluation were strictly conducted in accordance with the CLEAN. Under these conditions, CACLENS outperformed CLEAN in EC number prediction. In the comparison of reaction feasibility prediction performance, it is worth noting that the datasets of ESP and EnzRank were derived from multiple databases such as UniProt, Rhea, and BRENDA, covering a broader range of sources than those used for training and testing in CACLENS. However, since ESP and EnzRank only support enzyme–substrate pairs as input, the same datasets cannot be directly used for training and testing in CACLENS, and to date, no reaction feasibility prediction model has been reported to accept enzyme–reaction pairs as input. As a solution, we adopted a scheme heavily biased in favor of ESP and EnzRank. Specifically, 379 548 substrates and enzymes from the CACLENS test set were used as inputs. Owing to the large sample size, there was a potential risk of overlap with the training sets of ESP and EnzRank, which was not controlled for in the present analysis. In addition, prediction failures caused by model limitations were also disregarded (e.g., 2964 inputs could not be processed by ESP due to restrictions on the number of atoms in small molecules). As a result, even under such a biased strategy, CACLENS still exhibited superior performance. Remarkably, CACLENS was able to accurately predict the feasibility of enzyme‐catalyzed reactions even for enzymes it had never encountered before and for those with low sequence identity. Overall, by comparing with state‐of‐the‐art models, the results show that CACLENS demonstrates robust performance across all three tasks.

We assessed the performance of a standalone reaction feasibility prediction tower—by removing the contrastive loss, the EC number prediction tower, the reaction classification tower, and the CGC module—to reveal the improvement achieved by the multitask architecture. Except for these architectural removals, all other settings remained unchanged, and we adopted the combination of ProtT5 and MoLFormer as the encoders for proteins and small molecules, respectively. The results show that the multitask architecture substantially improved the prediction capability for reaction feasibility, with precision increasing from ≈0.81 to 0.97 and ROC‐AUC rising from ≈0.87 to ≈0.99 (Figure S16, Supporting Information).

Thanks to our adoption of the mixed precision computing mode of PyTorch,^[^ ^51^ ^]^ the training process only requires 1.5 days after the data encoding is completed. Notably, compared to 10 deep learning models for enzyme screening, CACLENS not only efficiently performs all three functions for enzyme screening but also validates its enzyme screening capability through experiments, while requiring minimal computational resources (Table S9, Supporting Information). Surprisingly, although CACLENS is primarily trained on a GPU, it has been successfully deployed on a lightweight Ubuntu server with an 8‐core CPU and 32GB of memory. Tests show that it only takes a few minutes to directly screen 1000 functional enzymes under pre‐encoding conditions. Furthermore, we provide an online server at https://ai.caclens.com/, designed to facilitate the use of CACLENS without the need for programming skills or specialized software (Figure S17A,B, Supporting Information). Researchers can submit data either individually or in batches using a CSV file. The prediction results can be downloaded directly from the website. Both the website and the GitHub scripts serve the same purpose and are based on the same underlying model. While the web server runs on a CPU rather than a GPU, it still produces identical results.

ZEN is one of the most common *Fusarium* mycotoxins contaminating cereal grains worldwide.^[^ ^52^ ^]^ The degradation of ZEN is significant to improve food safety and ensure human health. Enzymatic elimination of ZEN is highly specific and eco‐friendly. To experimentally evaluate the availability of CACLENS for ZEN degradation enzyme discovery, we screened and verified potential ZEN degradation enzymes based on CACLENS prediction. Although the catalytic efficiencies varied markedly among the candidates, we captured a robust enzyme ZD7 capable of efficiently hydrolyzing ZEN in buffer and food matrix scenarios, providing sufficient theoretical support in further application. This validated the effectiveness of CACLENS and provided promising enzymes to detoxify ZEN and its derivatives. The candidate ZD7 demonstrated remarkable catalytic activity toward ZEN and α‐ZOL. According to Uniport annotation, ZD7 was derived from the organism of *Venustampulla echinocandica* which was known for its antifungal properties on *Aspergillus fumigatus* and *Candida albicans*,^[^ ^53^ ^]^ while ZD7 is the first reported enzyme derived from *V. echinocandica* with ZEN degradation capability. Notably, Xu et al. reported that a known ZEN degradation enzyme ZHD101 preferred ZEN to α‐ZOL as its substrate. While our newly identified ZD7 has comparably high substrate preferences on ZEN and α‐ZOL. The catalytic activity of ZD7 on ZEN and ZOL is significantly different from previously identified ZHD101.^[^ ^54^ ^]^ Our study employed a novel computational workflow to directly screen degradation enzymes, transcending conventional microbial mining. This approach enabled large‐scale candidate evaluation and significantly accelerated the discovery timeline for novel enzymes.

Although CACLENS demonstrates strong robustness in multitask performance, it lacks interpretability, making it difficult to understand the reasoning behind its predictions, and neglects protein structure information. In the future, we intend to enhance CACLENS by incorporating advanced techniques to improve its interpretability, such as integrating methods like SHAP.^[^ ^55^ ^]^ Additionally, we plan to integrate protein structure data through tools like ESMFold^[^ ^56^ ^]^ or graph‐based models to better capture the relationship between sequence and 3D conformation. In summary, CACLENS is a multimodal, multitask framework that accelerates industrial enzyme discovery with reduced computational resources. By using ZEN as an example for CACLENS enzyme screening, we successfully predicted and identified degrading enzymes for ZEN. Moreover, CACLENS can be widely applied in various fields, including metabolic pathway design, environmental pollutant degradation, and industrial enzyme screen.

## 4 Experimental Section

### Dataset Construction

The substrate‐product‐enzyme triads were used for the reaction feasibility prediction task of CACLENS. To create substrate‐product‐enzyme triads, reaction data from Rhea^[^ ^25^ ^]^ were searched and enzyme sequences from UniProt were retrieved.^[^ ^11^ ^]^ For the reaction data in Rhea, reactions labeled as left‐to‐right (LR) or right‐to‐left (RL) were strictly followed according to the reaction rules, and only the unidirectional forms were retained during training. For reactions labeled as bidirectional (BI) or undefined direction (UN), both the forward and reverse reactions were used for training. For the reaction representations, the method proposed by Xing et al.,^[^ ^37^ ^]^ was followed using the Reaction Decoder Tool (RDT)^[^ ^57^ ^]^ based on the atom‐to‐atom mapping technique to process all the reaction data. Taking the ZEN degradation reaction as an example, the execution steps of the RDT‐based data cleaning procedure are shown in Figure S1 (Supporting Information).

The Rhea database only records reactions that enzymes can catalyze (positive data), while data for reactions that enzymes cannot catalyze (negative data) was difficult to acquire. A weighted random sampling strategy was adopted, following the method of Zhang et al.,^[^ ^58^ ^]^ as formulated below:

(1) $$\mathrm{Weigh}t_{p}=-\mathrm{lo}g_{10}\left(\frac{10}{X_{max}-X_{min}}\times \frac{X_{p}-X_{min}}{X_{max}-X_{min}}\right)$$

here, *X_p_* represents the number of corresponding reactions of the enzyme *p* in the positive dataset. *X_max_* is the largest number of reactions associated with any enzyme in the positive dataset, while *X_min_* is the minimum. Through this approach, negative data that was approximately ten times the size of the positive dataset was randomly sampled.

### Model Input Calculation

To represent substrate‐product‐enzyme triads, two widely used protein‐pretrained models were employed, ProtT5,^[^ ^30^ ^]^ ESM‐2 and the corresponding ProteinCLIP models to encode enzyme sequences. The pre‐trained UniMol^[^ ^16^ ^]^ model and MoLFormer^[^ ^31^ ^]^ model was used for feature extraction of reactants and products. Using four combinations of the four models as encoding models: ProtT5 + UniMol, ProtT5 + MoLFormer, ESM‐2 + UniMol, and ESM‐2 + MoLFormer. More details on the Model input calculation model and its implementation were provided in the Supporting Information.

### Multitask Model Construction

To build a model simultaneously predicting EC numbers, reaction types, and reaction feasibility, the seesaw phenomenon in MTL^[^ ^59^ ^]^ should be addressed. The CGC system developed by Tencent,^[^ ^59^ ^]^ which introduced a novel architecture that separates shared and task‐specific components was learned from. Based on this architecture, Wang et al.^[^ ^60^ ^]^ successfully implemented the simultaneous prediction of the *K_M_* and *k_cat_*.

Specifically, for the input reaction vector representation or enzyme vector representation, the model uses both shared experts and task‐specific expert modules to extract features. The gating network was adapted to different tasks, allowing for better control of the information flow, reduction in model parameters, and computational load. The output of the expert layer was synthesized through the gating system and input into the task‐specific tower layers (Figure 1C). This approach improves joint representation learning by efficiently managing task correlations, avoiding negative transfer, and enhanced multimodal learning across all three tasks (EC number prediction, reaction classification, and reaction feasibility prediction).

### Expert Layer and Gating Network

As shown in Figure 1C, two completely independent expert layers were used to process the vector representations of reactant‐product pairs and enzymes. Each expert layer consisted of a shared expert and two task‐specific expert modules (expert A and expert B). The gating network dynamically adjusted the contribution of the shared and expert modules to each prediction task. The gating network was a single‐layer feed‐forward network with SoftMax as the activation function.^[^ ^61^ ^]^ It functions as a selector by generating selection probabilities, which were then used to compute the weighted sum of the selected expert outputs. The CGC framework for the task *k* is as follows:

(2) $$w_{p}^{k}\left(x_{p}\right)=\mathrm{Softmax}\left(W_{pg}^{k}x_{p}\right)$$

(3) $$w_{r}^{k}\left(x_{r}\right)=\mathrm{Softmax}\left(W_{rg}^{k}x_{r}\right)$$

(4) $$g_{p}^{k}\left(x_{p}\right)=w_{p}^{k}\left(x_{p}\right)S_{p}^{k}\left(x_{p}\right)$$

(5) $$g_{r}^{k}\left(x_{r}\right)=w_{r}^{k}\left(x_{r}\right)S_{r}^{k}\left(x_{r}\right)$$

where *x_p_* (*x_protein_*) is the vector input for the protein, and *x_r_* (*x_reaction_*) is the vector input for the reactant‐product pair. $W_{pg}^{k}\in R^{\left(m_{pk}+m_{ps}\right)\times d_{p}}$ and $W_{rg}^{k}\in R^{\left(m_{rk}+m_{rs}\right)\times d_{r}}$ represent the parameter matrices for processing the protein or reactant‐product pair vectors, respectively. *m_pk_* and *m_rk_* represent the number of task *k*′s specific experts in protein and reaction expert layers, while *m_ps_* and *m_rs_* represent the number of shared experts. *d_p_* and *d_r_* represent the dimensions of the protein and reactant‐product pair vectors, respectively. $w_{p}^{k}\left(x_{p}\right)$ and $w_{r}^{k}\left(x_{r}\right)$ are the weighting functions for the protein expert layer and reaction expert layer, respectively, which calculate the weight vector of task k through the gating network. $S_{p}^{k}\left(x_{p}\right)$ and $S_{r}^{k}\left(x_{r}\right)$ are two selected matrices composed of the outputs of all expert modules, including shared experts and task k's specific experts:

(6) $$S_{p}^{k}\left(x_{p}\right)={\left[E_{\left(pk,1\right)}^{T},E_{\left(pk,2\right)}^{T},..,E_{\left(pk,m_{pk}\right)}^{T},E_{\left(ps,1\right)}^{T},E_{\left(ps,2\right)}^{T},..,E_{\left(ps,m_{ps}\right)}^{T}\right]}^{T}$$

(7) $$S_{r}^{k}\left(x_{r}\right)={\left[E_{\left(rk,1\right)}^{T},E_{\left(rk,2\right)}^{T},..,E_{\left(rk,m_{rk}\right)}^{T},E_{\left(rs,1\right)}^{T},E_{\left(rs,2\right)}^{T},..,E_{\left(rs,m_{rs}\right)}^{T}\right]}^{T}$$

### Tower Layer

The output of the expert layer will be passed through three towers for training on three different tasks:

(8) $$y^{ec}\left(x_{p}\right)=t^{ec}\left(g_{p}^{k=ec}\left(x_{p}\right)\right)$$

(9) $$y^{rcls}\left(x_{r}\right)=t^{rcls}\left(g_{r}^{k=rcls}\left(x_{r}\right)\right)$$

(10) $$y^{rpre}\left(x_{r},x_{p}\right)=t^{rpre}\left(g_{r}^{k=rpre}\left(x_{r}\right),g_{p}^{k=rpre}\left(x_{p}\right)\right)$$

where *y^ec^* (*x_p_*), *y^rcls^* (*x_r_*), and *y^rpre^* (*x_r_*,*x_p_*) represent the final predicted values for the EC number prediction, reaction classification, and reaction feasibility prediction tasks, respectively. *y^rpre^* (*x_r_*,*x_p_*) requires two inputs related to the reactant‐product pair and protein. *t^ec^*, *t^rcls^*, *t* ^*rpre*^represents the three towers that perform the specific tasks. $g_{p}^{k=ec}\left(x_{p}\right)$, $g_{r}^{k=rcls}\left(x_{r}\right)$, $g_{r}^{k=rpre}\left(x_{r}\right)$, $g_{p}^{k=rpre}\left(x_{p}\right)$ represents inputs of the tower layer for specific task.

The description of each tower is as follows:

### EC Number Prediction Tower

The EC Prediction Tower consisted of an optional protein expert module, followed by fully connected layers, Layer Normalization, ReLU function (as follows), and Dropout. It specifically accepts the input $g_{p}^{k=ec}\left(x_{p}\right)$.

(11) $$\mathrm{ReLU}\left(x\right)=max\left(0,x\right)$$

In this tower, the contrastive learning training method proposed by Yu et al.,^[^ ^6^ ^]^ was referred to, where proteins were mapped to an embedding space through training, and the cosine similarity between proteins reflects the similarity between samples. Instead of randomly choosing negative samples, those with a smaller Euclidean distance from the anchor sequence are prioritized. This approach provides more challenging negative samples, improving training efficiency and helping CACLENS better handle imbalanced data. The loss function for training is as follows:

(12) $$\mathrm{Loss}=\sum_{e\in E}\frac{-1}{\left|P\left(e\right)\right|}\sum_{z_{p}\in P\left(e\right)}\;log\frac{\exp\left(z_{e}\cdot z_{p}/\tau \right)}{\sum_{z_{a}\in A\left(e\right)}\;\exp\left(z_{i}\cdot z_{a}/\tau \right)}$$

where P(e) represents the set of positive samples that belong to the same EC category e as the anchor *z_e_*, z_p_εP(e) is the embedding vector of a positive sample in contrastive learning. N(e) denotes the set of negative samples selected through hard negative mining, consisting of proteins with EC numbers different from the anchor protein. A(e)=P(e)∪N(e) represents the union of the positive and negative sample sets. In the inference stage, the greedy method was used, which selected EC numbers with the maximum separation (outstanding) in terms of the pairwise distance to the query sequence.

### Reaction Classification Tower

The Reaction Classification Tower accepted $g_{r}^{k=rcls}\left(x_{r}\right)$ as input. The main architecture of this Tower and the loss function were the same as those of the EC Number Prediction Tower. However, the reaction classification task is different from the EC number prediction for enzymes. During the training phase, a completely random negative sample sampling mechanism was used. The vectors output by the trained Reaction Classification Tower for both the training and test sets were treated as reaction fingerprints. During the inference phase, these fingerprints are inferred through either logistic regression or Euclidean distance calculation.

### Reaction Prediction Tower

The Reaction Prediction Tower required $g_{r}^{k=rpre}\left(x_{r}\right)$ and $g_{p}^{k=rpre}\left(x_{p}\right)$ as inputs to predict the reaction feasibility. These inputs were processed further through the multi‐head cross‐attention mechanism, a technique grounded in the attention mechanism, and the formula is as follows:

(13) $$Attention\left(Q,K,V\right)=\frac{\exp\left(\frac{QK^{T}}{\sqrt{d}}\right)}{\sum_{i}\exp\left(\frac{QK^{T}}{\sqrt{d}}\right)}V$$

The attention mechanism works by calculating the similarity between the query vector Q and the key vector K (usually through a dot product). This produces a weighted average of the value vectors V, with the weights determined by the similarity scores. $Q\in R^{d_{q}}$ is the query vector, with dimensionality *d_q_*, $K\in R^{d_{k}}$ is the key vector, with dimensionality *d_k_*, $V\in R^{d_{v}}$ is the value vector, with dimensionality *d_v_*, $\sqrt{d}$ is the scaling factor for the key vector's dimension.

Building upon the attention mechanism, the cross‐attention mechanism^[^ ^62^ ^]^ was used to capture the interactions between the protein and the reaction:

(14) $$\begin{array}{cc} CrossAtt_{protein} & =softmax\left(\frac{Q_{P}K_{P}^{T}}{\sqrt{d_{k_{P}}}}\right)V_{P}\\ & =softmax\left(\frac{\left(XW_{P}^{Q}\right){\left(YW_{P}^{K}\right)}^{T}}{\sqrt{d_{k_{X}}}}\right)\left(YW_{P}^{V}\right) \end{array}$$

(15) $$\begin{array}{cc} CrossAtt_{reaction} & =softmax\left(\frac{Q_{R}K_{R}^{T}}{\sqrt{d_{k_{R}}}}\right)V_{R}\\ & =softmax\left(\frac{\left(YW_{R}^{Q}\right){\left(XW_{R}^{K}\right)}^{T}}{\sqrt{d_{k_{R}}}}\right)\left(XW_{R}^{V}\right) \end{array}$$

(16) $$softmax\left(x_{i}\right)=\frac{e^{x_{i}}}{\sum_{i}e^{x_{i}}}$$

(17) $$\mathrm{MultiHead}(\mathrm{CrossAtt})=\mathrm{Concat}(\mathrm{hea}d_{1},head_{2},,head_{h})W^{O}$$

In the cross‐attention mechanism for protein, the query matrix *Q_P_* is computed from $g_{p}^{k=rpre}\left(x_{p}\right)$, while the key matrix *K_P_* and value matrix *V_P_* are computed from $g_{r}^{k=rpre}\left(x_{r}\right)$. For the cross‐attention mechanism applied to reaction, the roles of the inputs are reversed. The six matrices $W_{P}^{K}$, $W_{P}^{V}$ $W_{P}^{Q}$, $W_{R}^{K}$, $W_{R}^{V}$, and $W_{R}^{Q}$ are learnable parameters, and $d_{k_{P}}$ and $d_{k_{R}}$ denote the dimensions of *K_P_* and *K_R_*, respectively.

In the Reaction Prediction Tower, Leaky ReLU was used as an activation function to address the “dying units” problem. Leaky ReLU allows a small, non‐zero gradient to flow for negative input values, ensuring that the unit remains active during training and does not become inactive:

(18) $$\mathrm{LeakyReLU}\left(x\right)=\{\begin{array}{c} x\;\;if\;\;x>0\\ \alpha x\;\;\;\;if\;\;x\leq 0 \end{array}$$

where α is a hyperparameter, which was set to 0.01.

Following the cross‐attention, $g_{p}^{k=rpre}\left(x_{p}\right)$ and $g_{r}^{k=rpre}\left(x_{r}\right)$ were expanded into 1D vectors. After concatenation, they passed through the final fully connected layer, producing a 2D output vector. A smoothed label loss function was used during the training phase:

(19) $$\mathrm{Loss}=-\frac{1}{N}\sum_{i=1}^{N}\left[\left(\beta \cdot y\right)\log\left(p\right)+\left(1-\left(1-\beta \right)\cdot y\right)\log\left(1-p\right)\right]$$

where N represents the batch size, *y* denotes the ground truth, and *p* is the predicted probability. β refers to the label smoothing parameter, which is set to 0.1.

### Model Optimization and Parameters

Xavier initialization was used to set the initial weights of the neural network, ensuring that the variance of the outputs between layers is maintained, which prevented issues such as vanishing or exploding gradients. For a layer with *n_in_* input units and *n_out_* output units, the description of Xavier initialization was as follows:

(20) $$W∼U\left(-\sqrt{\frac{6}{n_{in}+n_{out}}},\sqrt{\frac{6}{n_{in}+n_{out}}}\right)$$

where W is the weight matrix, U denotes the uniform distribution.

The optimization method of Zhang et al.^[^ ^58^ ^]^ was referred to, and RAdam^[^ ^63^ ^]^ was used as the optimizer, along with the LookAhead^[^ ^64^ ^]^ mechanism. RAdam adjusted the learning rate by introducing a "rectification factor," making the training process more stable in the early stages. The main formula of RAdam was as follows:

(21) $$m_{t}=\beta_{1}m_{t-1}+\left(1-\beta_{1}\right)g_{t}$$

(22) $$v_{t}=\beta_{2}v_{t-1}+\left(1-\beta_{2}\right)g_{t}^{2}$$

where *m_t_* is the first moment estimate (momentum), which is the weighted average of the gradients; *v_t_* is the second moment estimate (the weighted average of the squared gradients), used to estimate the variance of the gradients; *g_t_* is the current gradient, and β_1_ and β_2_ are the decay rates for the first and second moments, respectively.

RAdam introduced correction factors (${\hat{m}}_{t}$ and ${\hat{v}}_{t}$) to compensate for the bias in the first and second moment estimates during the early stages of training:

(23) $${\hat{m}}_{t}=\frac{m_{t}}{1-\beta_{1}^{t}}$$

(24) $${\hat{v}}_{t}=\sqrt{\frac{v_{t}}{\left(1-\beta_{2}^{t}\right)}}$$

where $\beta_{1}^{t}$ and $\beta_{2}^{t}$ represent the exponential decay of the first and second moment estimates' decay rates at time step *t*. Then, RAdam used these corrected moment estimates to compute the adaptive learning rate and update the model parameters, as shown in the following formula:

(25) $$\rho_{\infty }=2/\left(1-\beta_{2}\right)-1$$

(26) $$r_{t}=\sqrt{\frac{\left(\rho_{t}-4\right)\left(\rho_{t}-2\right)\rho_{\infty }}{\left(\rho_{\infty }-4\right)\left(\rho_{\infty }-2\right)\rho_{t}}}$$

(27) $$\theta_{t}=\left\{\begin{array}{c} \theta_{t-1}-\;\;\frac{\alpha_{t}r_{t}{\hat{m}}_{t}}{{\hat{\nu }}_{t}}\;\;if\;\;\rho_{t}>4\\ \theta_{t-1}-\;\;\alpha_{t}{\hat{m}}_{t}\;\;if\;\;\rho_{t}\leq 4 \end{array}\right.$$

where α_*t*_ is the step size, ρ_∞_ represents the maximum length of the approximated moving average. ρ_*t*_ represents the current length of the approximated simple moving average (SMA), If ρ_*t*_ > 4, the variance correction term will be used.

The core of lookahead was that during training, the model has two sets of weights: fast weights (θ) and slow weights (ϕ). In each iteration, the fast weights were updated k times using the optimizer, and then the slow weights were updated to correct them. RAdam was used to perform k updates on each batch of data, where the *i*‐th step (i∈{1,2,..,k}) of the fast weight update is:

(28) $$\theta_{t,i+1}=\theta_{t,i}+\mathrm{RAdam}\left(L,\theta_{t,i-1},d\right)$$

where RAdam is the inner loop optimizer, L is the objective function, θ_*t*,*i*_ is the current fast weights, and *d* is the sample minibatch of data. After completing k updates of the fast weights, the slow weights are updated once. The update of the slow weights was done by linearly interpolating them toward the final fast weights:

(29) $$\phi_{t}=\phi_{t-1}+\alpha \left(\theta_{t,k}-\phi_{t-1}\right)$$

where ϕ_*t*_ is the current slow weights, θ_*t*,*k*_ is the fast weights at the end of the inner loop (after k updates), α is the step size for updating the slow weights, controlling the amount of movement toward the fast weights. Through this update, the slow weights are adjusted smoothly in the direction of the fast weights, avoiding the drastic fluctuations of the fast weights.

### Evaluation Metrics

The model involved three different classification tasks: EC number prediction, reaction classification, and reaction feasibility prediction, and its evaluation adopts standard classification metrics, including TPR, FPR, Precision, Recall, ROC‐AUC, PRC‐AUC, F1 Score, and MCC, with detailed definitions and explanations provided in the Supporting Information.

### Screening of Candidate Enzymes for ZEN Degradation

The reactants and products of the ZEN degradation reaction were obtained after applying the RDT‐based data cleaning procedure. Meanwhile, 100 000 unannotated candidate enzyme sequences were downloaded from UniProt. The Reaction Classification Tower of CACLENS‐EF (fine‐tuned on the EC‐Reaction dataset, see Supporting Information) received the reactants and products as input and predicted the relevant EC number. CACLENS‐EF initially identified the EC number of the known ZEN‐degrading enzyme (lactone bond hydrolyses) as 3.1.1.‐. Then, the EC Number Prediction Tower of CACLENS‐EF performed EC number prediction on 100 000 unannotated candidate enzymes and used EC 3.1.1.‐ as a criterion for preliminary screening, narrowing down the candidate enzyme pool to 10 000. Finally, the Reaction Prediction Tower of CACLENS‐TF evaluated the feasibility of enzyme‐catalyzed reactions using a 0–1 scoring system, where a score closer to 1 indicates higher reaction feasibility.

### Evaluation of the Catalytic Activity of Candidate Enzymes

According to the predicted probability, 10 potential enzymes were selected for further expression and activity assay. The gene sequences of candidate enzymes were directly synthesized and cloned into pET‐28 vector with His‐Tag by Tsingke Biotech (Beijing). The constructed plasmids with candidate genes were expressed by prokaryotic *E. coli* (DE3). All candidates were successfully expressed in pre‐experiment, and the candidate enzymes were purified by Ni‐NTA His‐Tag Purification Agarose.

To test the catalytic activity of purified enzymes, the mycotoxin ZEN (Final concentration 1000 ng mL^−1^) and 1 ug of each enzyme were added to 1 mL of Tris‐HCL buffer for reaction. After 1 h incubation at 37 °C, 1 mL of methanol was added to stop the reaction. The mixture was shaken and centrifuged at 13 000 g for 10 min. Finally, the supernatant was filtered using a 0.22 µm filter before HPLC analysis. Mycotoxin products of ZEN analysis were performed according to the previously developed HRMS method. The degradation experiments were independently performed with three repeats in each group.

### Web Server

The CACLENS website was created on a network server with an 8‐core CPU and 32 GB of RAM running Ubuntu 22.04. The website implementation adheres to the latest web standards, including HTML 5 and CSS 3. The front‐end interface was built using a Progressive JavaScript Framework, while the back‐end development was carried out using Python and the Django application framework.

## Conflict of Interest

The authors declare no conflict of interest.

## Author Contributions

A.W., and Y.T. designed the research. X.Y. developed and tested the deep‐learning model. Y.T., Y.T., and H.L. performed the experiments and provided experimental resources. X.Y., Y.T., G.Z., and A.W. wrote the paper. All authors approved the final paper.

## Supporting information



Supporting Information

## Data Availability

The data used in this study were obtained from two databases, with the most recent version available as of April 2025. The enzyme‐substrate pair dataset used for model training was sourced from Rhea (https://www.rhea‐db.org/), and the amino acid sequences of enzymes were retrieved from UniProt (https://www.uniprot.org/). Five benchmark datasets – split10, split30, split50, split70, and split100 – were sourced from https://github.com/tttianhao/CLEAN/tree/main/app/data. The Schneider 50k ^34^ dataset was downloaded from https://github.com/rxn4chemistry/rxnfp/tree/master/data. The data related to CACLENS can be downloaded from https://zenodo.org/records/16979412.
