# Supplementary material for: CACLENS: A Multitask Deep Learning System for Enzyme Discovery
Source: Adv Sci (Weinh). 2025 Dec 8;13(9):e18063. doi: 10.1002/advs.202518063 (PMC12904076; doi:10.1002/advs.202518063)
Supplement: Supplementary file 1 — Supporting Information [file ADVS-13-e18063-s001.docx]

1. **Supplementary methods**

**1.1** **Encoding Models**

**Enzyme encoding**

ProtT5 is a pretrained model based on T5-3B for protein sequence analysis, using a masked language modeling (MLM) objective with 24 layers and 3B parameters. The half-precision model is available on the Hugging Face Hub ^1^ at https://huggingface.co/Rostlab/prot_t5_xl_half_uniref50-enc. ESM-2 (Evolutionary Scale Modeling v2) is a protein language model developed by Meta AI, which uses a Transformer architecture similar to those in natural language processing, training on amino acid sequences through a self-supervised MLM. Pre-trained checkpoints available in their codebase at <https://github.com/facebookresearch/esm> (36-layer model: esm2_t36_3B_UR50D).

The encoding process by ProtT5 or ESM-2 involves tokenizing and position-encoding each amino acid. During the encoding process, all protein sequence lengths are standardized to 1500. Sequences shorter than 1500 were padded with padding tokens (e.g., `[PAD]`), while those exceeding 1500 were truncated. The protein sequence was transformed into an $n\times m$ dimensional vector using ProtT5 or ESM-2, where $\text{n}$ represents the amino acid sequence length and $\text{m}$ is the $\text{1024}$-dimensional hidden vector size. After performing mean pooling on the final output vectors, a $\text{1024}$-dimensional representation was obtained for each enzyme.

ProteinCLIP is a model that enhances protein language models (pLMs) ^2^ by aligning protein amino acid sequences with their functional descriptions through contrastive learning. To embed protein with function, we use the corresponding version of the ProteinCLIP model for the output vectors of ProtT5 or ESM-2. Pre-trained checkpoints available in the codebase at <https://github.com/wukevin/proteinclip>.

**Reactant-product pair encoding**

Uni-Mol is a 3D molecular representation framework based on Transformer architecture, trained on 209M molecular conformations and 3M protein pockets. The model is available on GitHub: <https://github.com/dptech-corp/Uni-Mol>. MoLFormer, a transformer-based model trained on over 1.1 billion molecules, learns molecular representations from SMILES sequences and can partially recover 3D molecular relationships via self-supervised learning. The pre-trained model version is available on Hugging Face: <https://huggingface.co/ibm-research/MoLFormer-XL-both-10pct>.

After cleaning the reaction data using the RDT process, the SMILES sequences of the reactants and products are input into the Uni-Mol or MoLFormer model, respectively. The outputs of the models are mapped to 512-dimensional vectors through a fully connected layer. The reactants and products each obtain vectors $r$ and $p$, respectively. For a given chemical reaction, we concatenate the embedding representations of the reactant and product to obtain a $2\times512$ dimensional vector, as shown in the formula. The vector $v$ obtained from concatenation was used for the subsequent task.

$$r\in\mathbb{R}^{1\times512}$$

$$p\in\mathbb{R}^{1\times512}$$

$$v=\left[ \begin{aligned} r \\ p \end{aligned} \right]\in\mathbb{R}^{2\times512}$$

**1.2 Evaluation Metrics**

The evaluation metrics used by CACLENS are as follows:

$$TPR=\frac{TP}{TP+FN}$$

$$FPR=\frac{FP}{FP+TN}$$

$$Precision=\frac{TP}{TP+FP}$$

$$Recall=\frac{TP}{TP+FN}$$

$$ROC-AUC=\int_{0}^{1} TPR(FPR)dFPR$$

$$PRC-AUC=\int_{0}^{1} Precision(Recall)dRecall$$

$$F1=\frac{2\times Precision\times Recall}{Precision+Recall}$$

$$MCC=\frac{TP\times TN-FP\times FN}{\sqrt{(TP+FP)(TP+FN)(TN+FP)(TN+FN)}}$$

Where $TP$ (True Positives) refers to correctly predicted positive samples, while $FP$ (False Positives) refers to samples incorrectly predicted as positive. $TN$ (True Negatives) refers to correctly predicted negative samples, and $FN$ (False Negatives) refers to samples incorrectly predicted as negative. $ROC-AUC$ ranges from 0 to 1, with a value closer to 1 indicating better class separation. $PRC-AUC$ also ranges from 0 to 1, where a value closer to 1 indicates better performance on imbalanced datasets. F1 Score ranges from 0 to 1, with higher values indicating a better balance between precision and recall. Matthews Correlation Coefficient (MCC) ranges from -1 to 1, where 1 indicates perfect prediction, -1 indicates completely wrong predictions, and 0 suggests random guessing.

For the Schneider 50k dataset, to compare the results on the imbalanced classification test set, we referred to the Confusion Entropy method ^3^ used by Schwaller et al. ^4^, which is calculated as follows:

$$P_{i,j}^{j}=\frac{\mathrm{Matrix}(i,j)}{\sum_{k=1}^{|C|} (\mathrm{Matrix}(j,k)+\mathrm{Matrix}(k,j))},$$

$$P_{i,j}^{i}=\frac{\mathrm{Matrix}(i,j)}{\sum_{k=1}^{|C|} (\mathrm{Matrix}(i,k)+\mathrm{Matrix}(k,i))}$$

$$\mathrm{CEN}_{j}=-\sum_{k=1,k\neq j}^{|C|} \left( P_{j,k}^{j}\log_{2(|C|-1)}\left( P_{j,k}^{j} \right)+P_{k,j}^{j}\log_{2(|C|-1)}\left( P_{k,j}^{j} \right) \right)$$

$$P_{j}=\frac{\sum_{k=1}^{|C|} (\mathrm{Matrix}(j,k)+\mathrm{Matrix}(k,j))}{2\sum_{k,l=1}^{|C|} \mathrm{Matrix}(k,l)}$$

$$\mathrm{CEN}=\sum_{j=1}^{|C|} P_{j}\mathrm{CEN}_{j}$$

The overall MCC ^5,6^, which is especially effective for imbalanced datasets, is defined as follows:

$$\mathrm{cov}(X,Y)=\sum_{i,j,k=1}^{|C|} (\mathrm{Matrix}(i,i)\mathrm{Matrix}(k,j)-\mathrm{Matrix}(j,i)\mathrm{Matrix}(i,k))$$

$$\mathrm{cov}\left( X,X \right)=\sum_{i=1}^{\left| C \right|} \left[ \left( \sum_{j=1}^{\left| C \right|} \mathrm{Matrix}\left( j,i \right) \right)\left( \sum_{k,l=1,k\neq i}^{\left| C \right|} \mathrm{Matrix}\left( l,k \right) \right) \right]$$

$$\mathrm{cov}(Y,Y)=\sum_{i=1}^{|C|} \left[ \left( \sum_{j=1}^{|C|} \mathrm{Matrix}(i,j) \right)\left( \sum_{k,l=1,k\neq i}^{|C|} \mathrm{Matrix}(k,l) \right) \right]$$

$$\mathrm{MCC}=\frac{\mathrm{cov}(X,Y)}{\sqrt{\mathrm{cov}(X,X)\times\mathrm{cov}(Y,Y)}}$$

**1.3 Reaction Classification Tower Fine-tuning**

Since the Schneider 50k dataset only contains reaction classification information but lacks EC numbers of enzymes required for catalyzing chemical reactions, we curated the Rhea and UniProt split100 datasets to construct the EC-Reaction dataset, supplementing the missing EC-related information.

Additionally, we employed a new contrastive learning sampling method, distinct from the one used in the Schneider 50k dataset. This method is based on an EC-level matching mechanism, dynamically selecting positive samples and negative samples according to different EC levels (from level 4 to level 1).

Specifically, the method first group reaction embeddings based on their EC numbers. During positive sample selection, the system filters out samples that match the anchor reaction based on the EC-level matching mechanism while ensuring duplicate samples are removed. For negative sample selection, the system first extracts the first digit of the EC number of the anchor sample, then selects reactions with a different first-digit EC number as negative samples, ensuring they do not overlap with the anchor reaction. This approach enhances the functional distinction between negative and anchor samples while maintaining diversity within the negative sample set.

First, we used the entire Schneider 50k dataset as the training set, applying the same approach used in the Tower layer section of methods, allowing CACLENS to fully learn different reaction types. Then, we split the EC-Reaction dataset, with 80% as the training set and 20% as the test set. We then fine-tuned the trained CACLEANS Reaction Classification Tower for 5 epochs using a learning rate of 8.0 × 10⁻⁵.

During the inference phase, the fine-tuned Reaction Classification Tower converts the input reaction into a feature vector and compares it with the precomputed reaction embeddings from the training set of EC-Reaction dataset using cosine similarity, ultimately selecting the top 5 most similar EC numbers as predictions. To evaluate the model, we match the predicted 5 EC numbers with the ground truth EC number at four hierarchical EC levels. Since the primary goal of the Reaction Classification Tower is to narrow down enzyme selection, we assess its performance by calculating the number of correctly matched EC numbers in the test set of EC-Reaction dataset, which serves as the accuracy metric.

1. **Supplementary Figure**


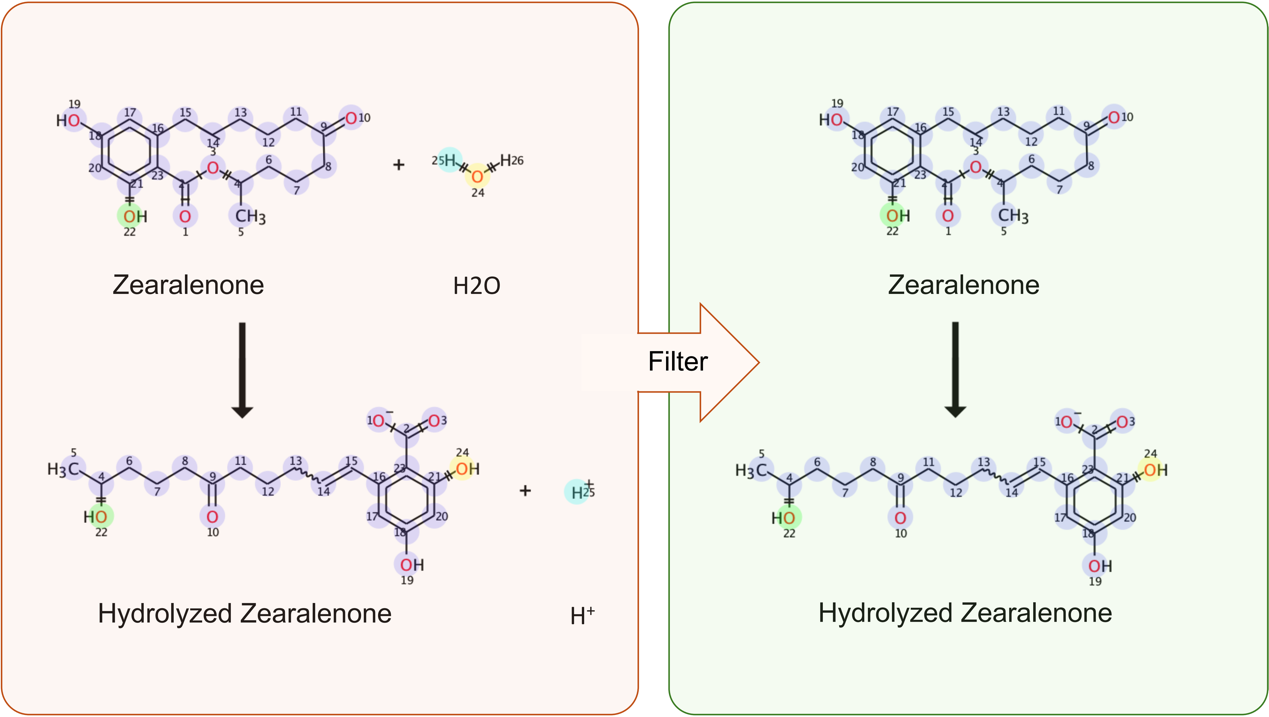


**Figure S1.** The process of cleaning reaction data. Atom-to-atom mapping can extract substrate-product pairs from complete reaction data.


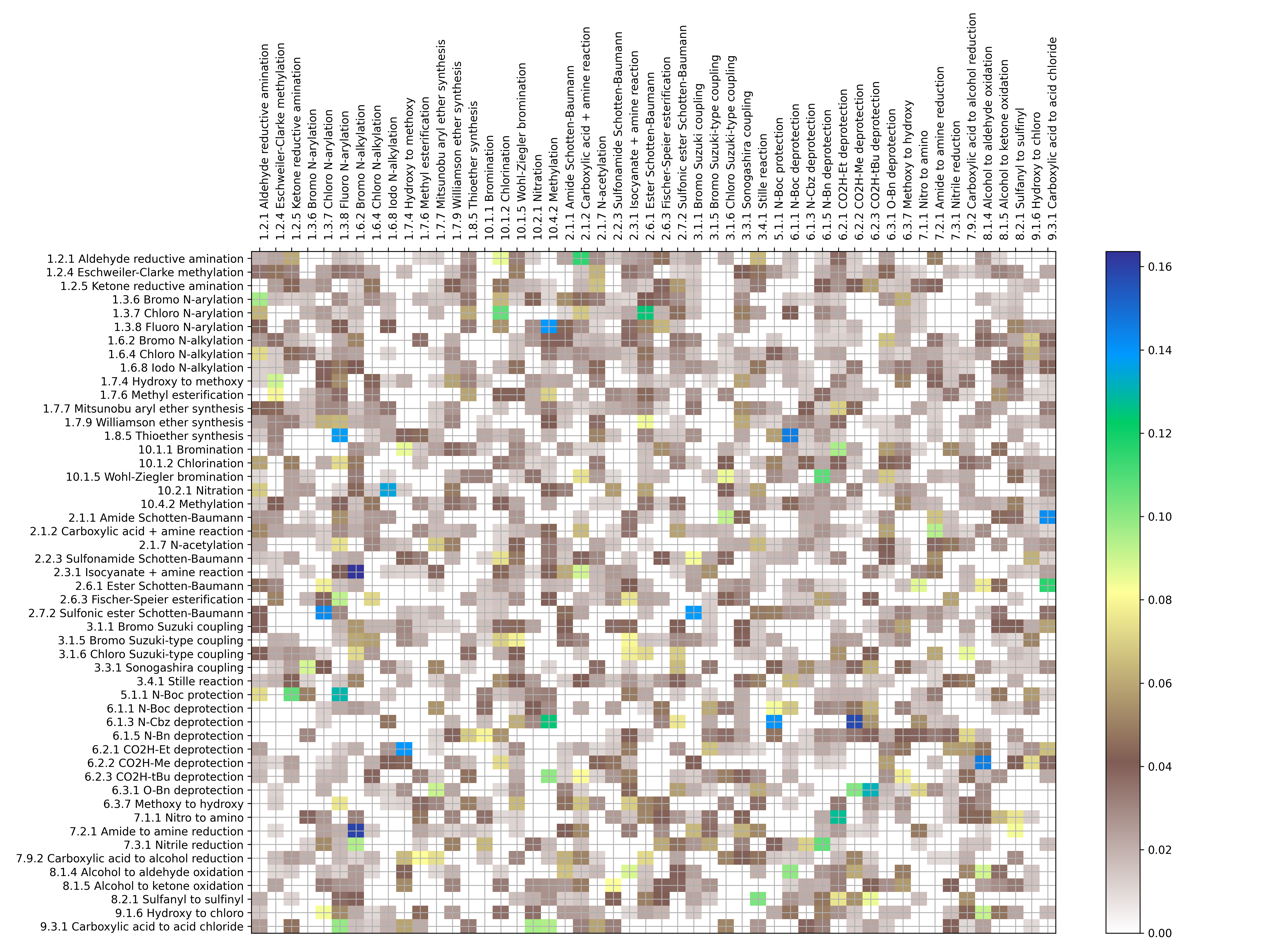


**Figure S2.** Confusion matrix for Random classification (predicting after shuffling the labels of the test data from CACLENS-EF).


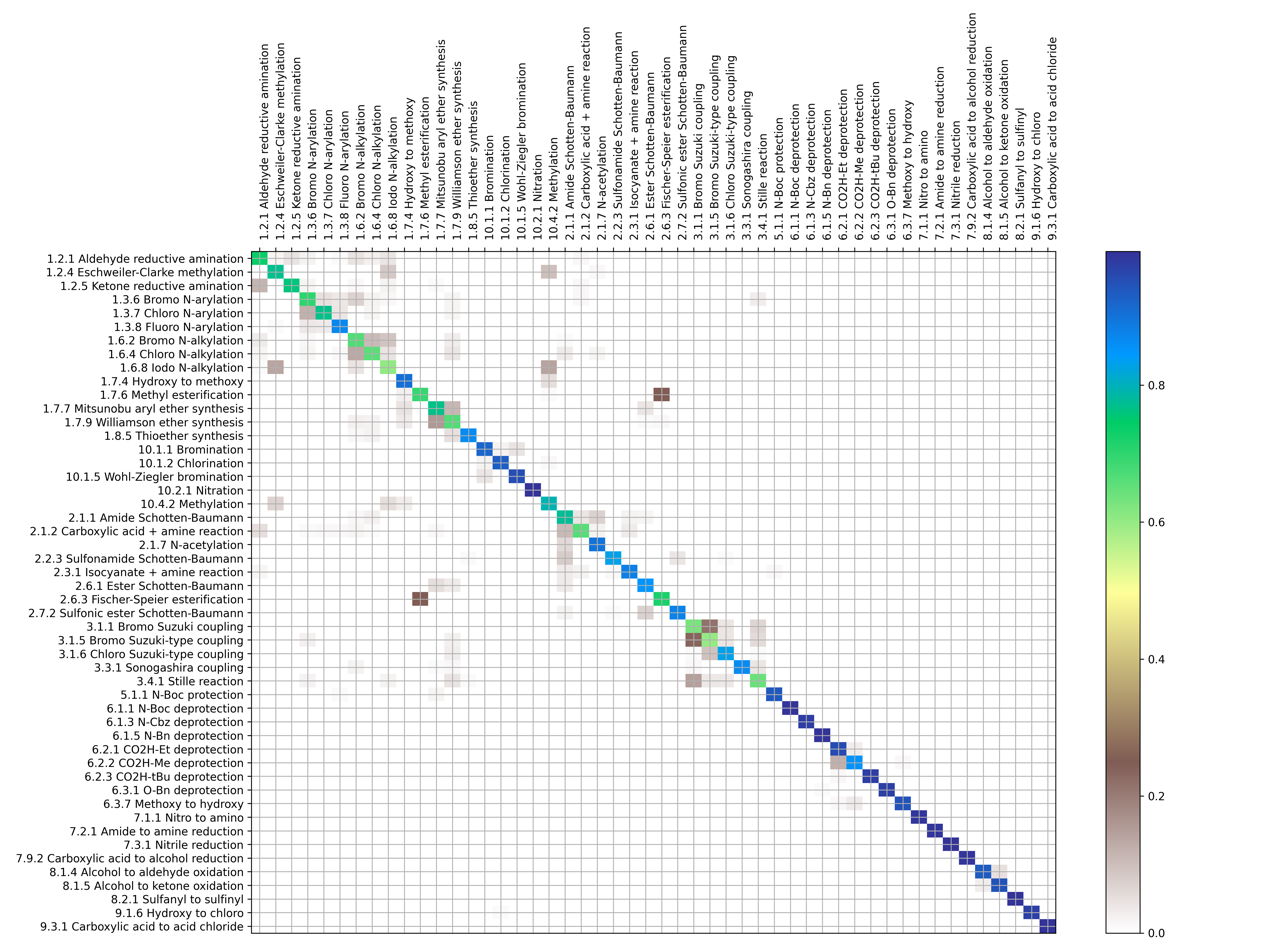


**Figure S3.** Confusion matrix for CACLENS-EU (logistic regression).


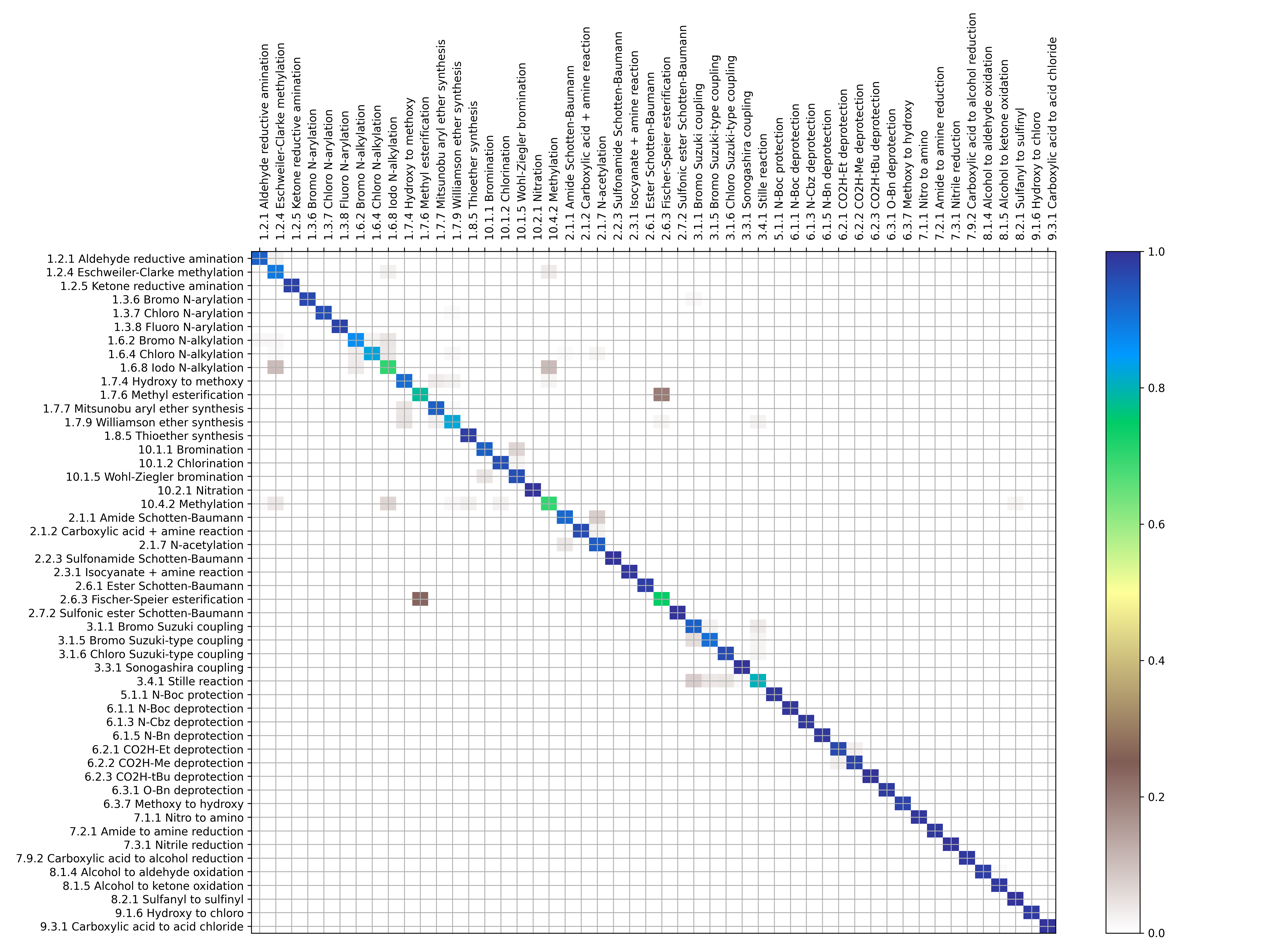


**Figure S4.** Confusion matrix for CACLENS-EF (logistic regression).

**
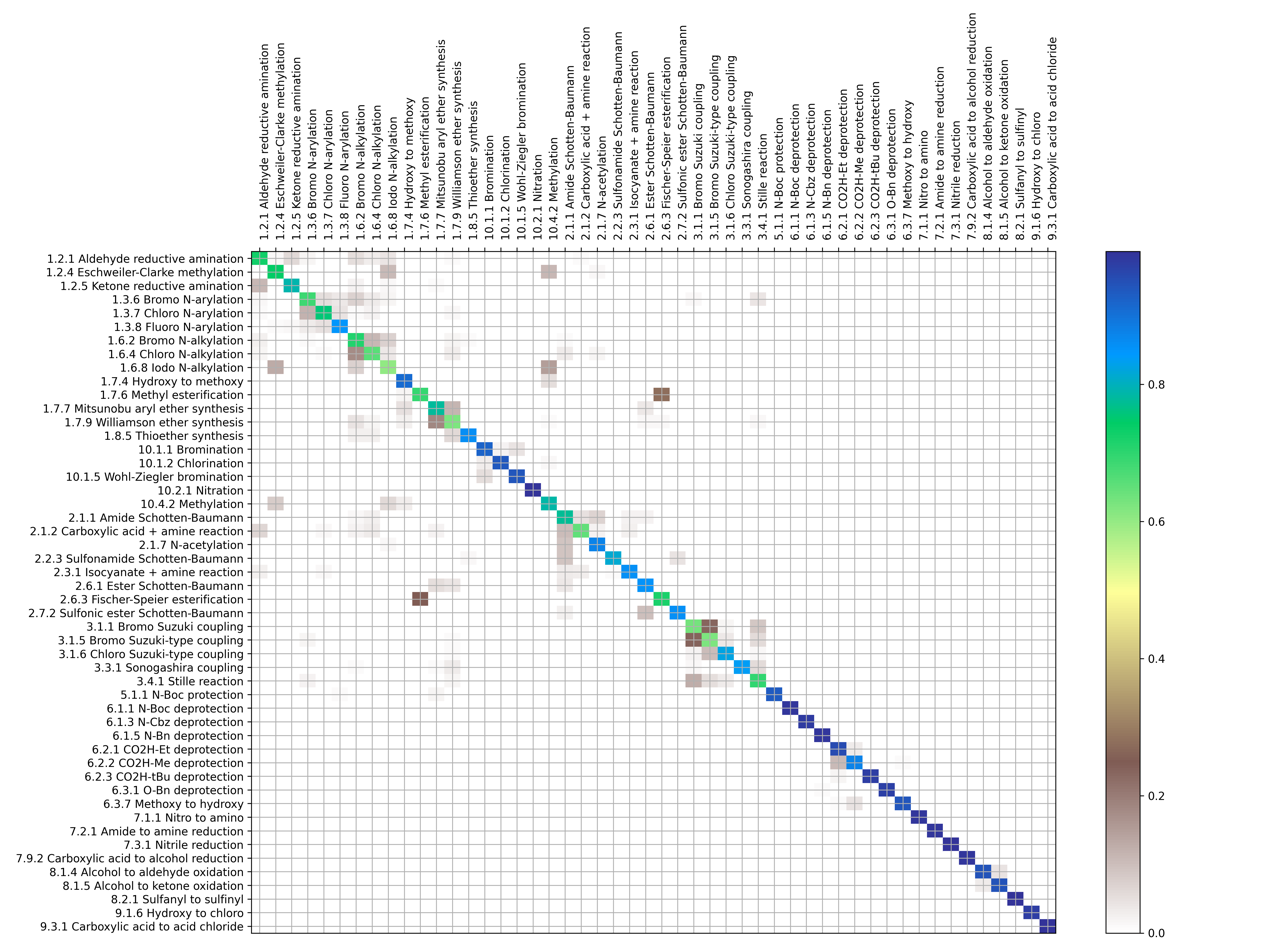
**

**Figure S5.** Confusion matrix for CACLENS-EU (Euclidean distance).


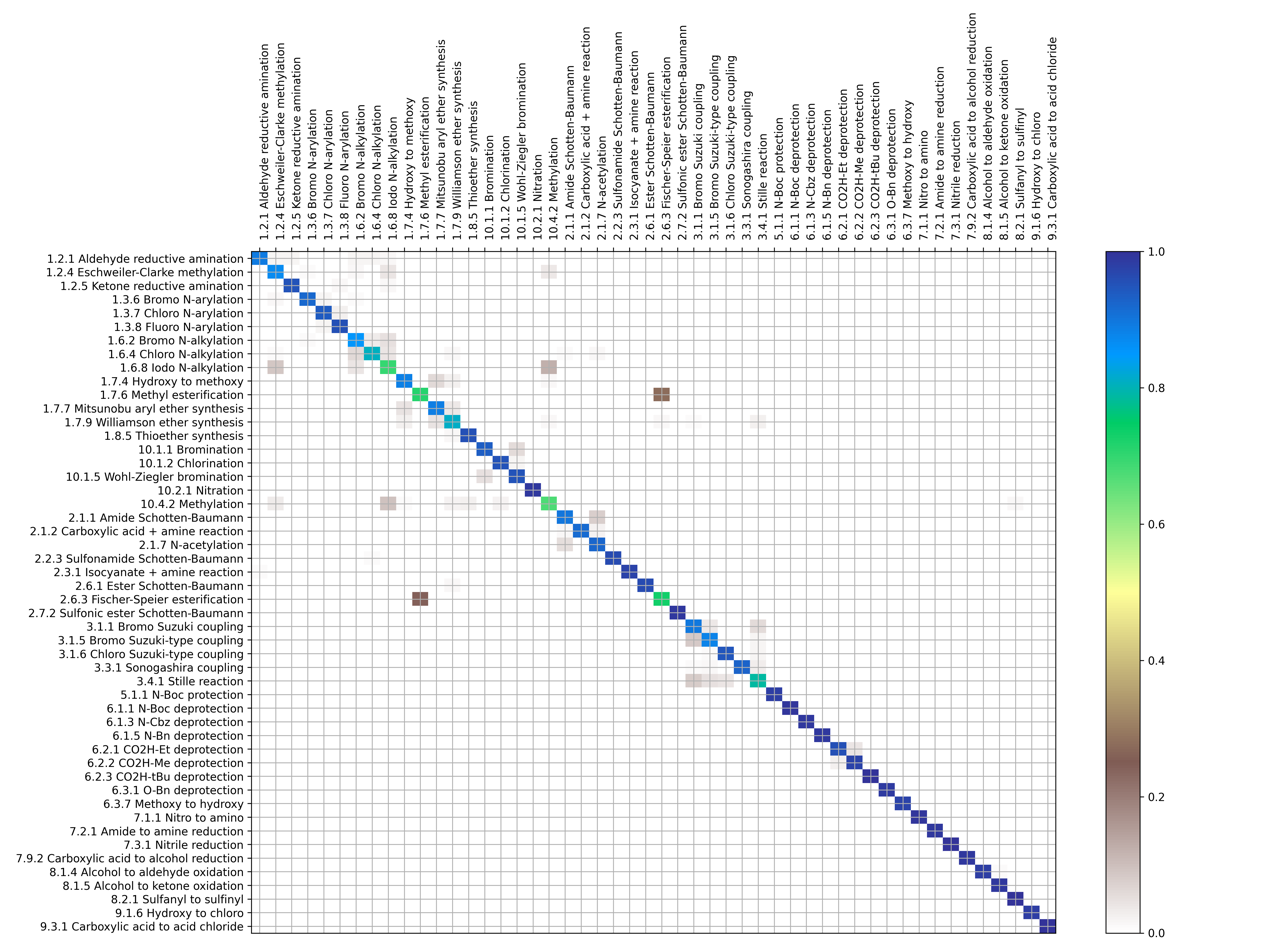


**Figure S6.** Confusion matrix for CACLENS-EF (Euclidean distance).


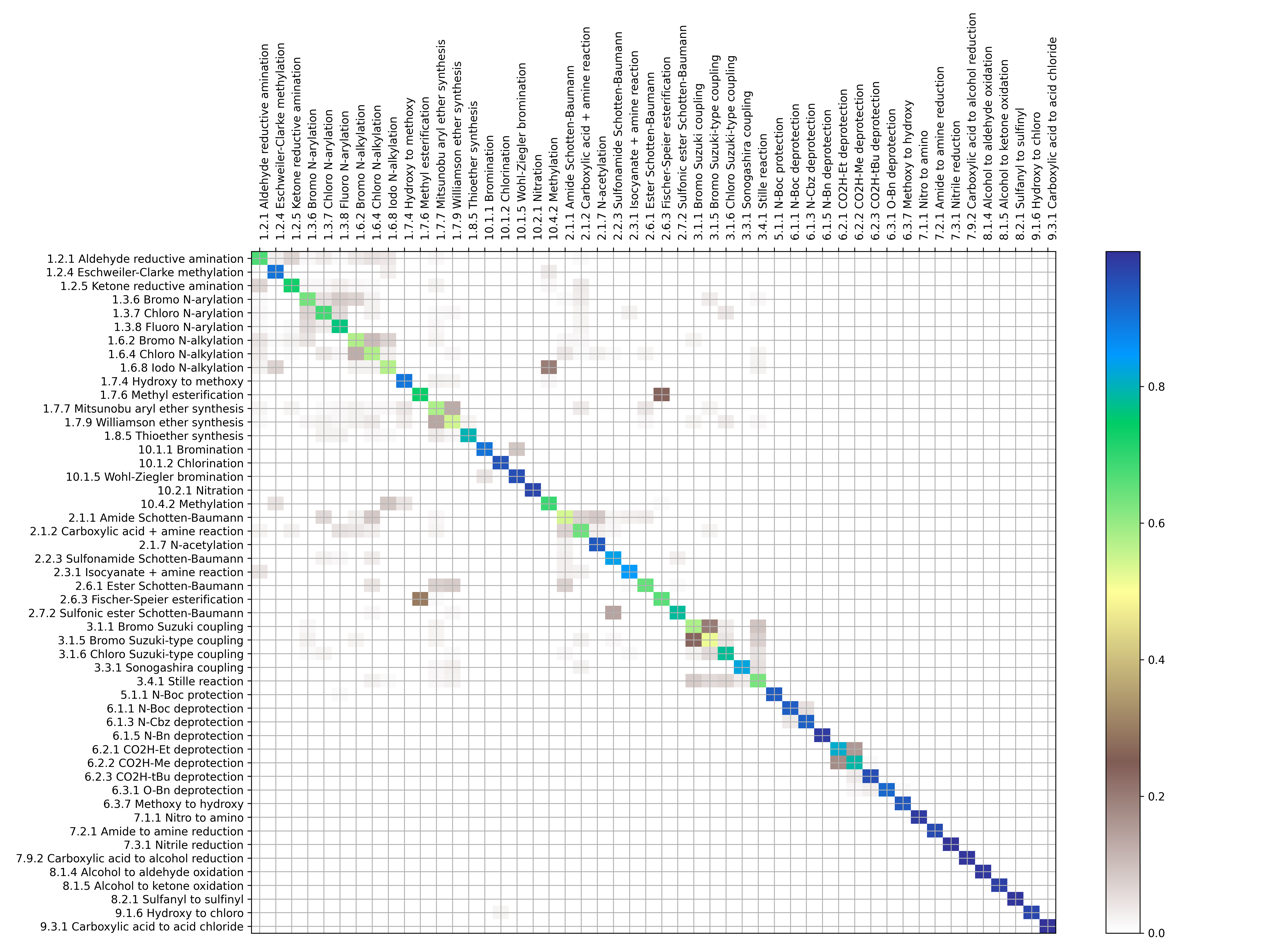


**Figure S7.** Confusion matrix for *rxnfp* (pretrained)

**
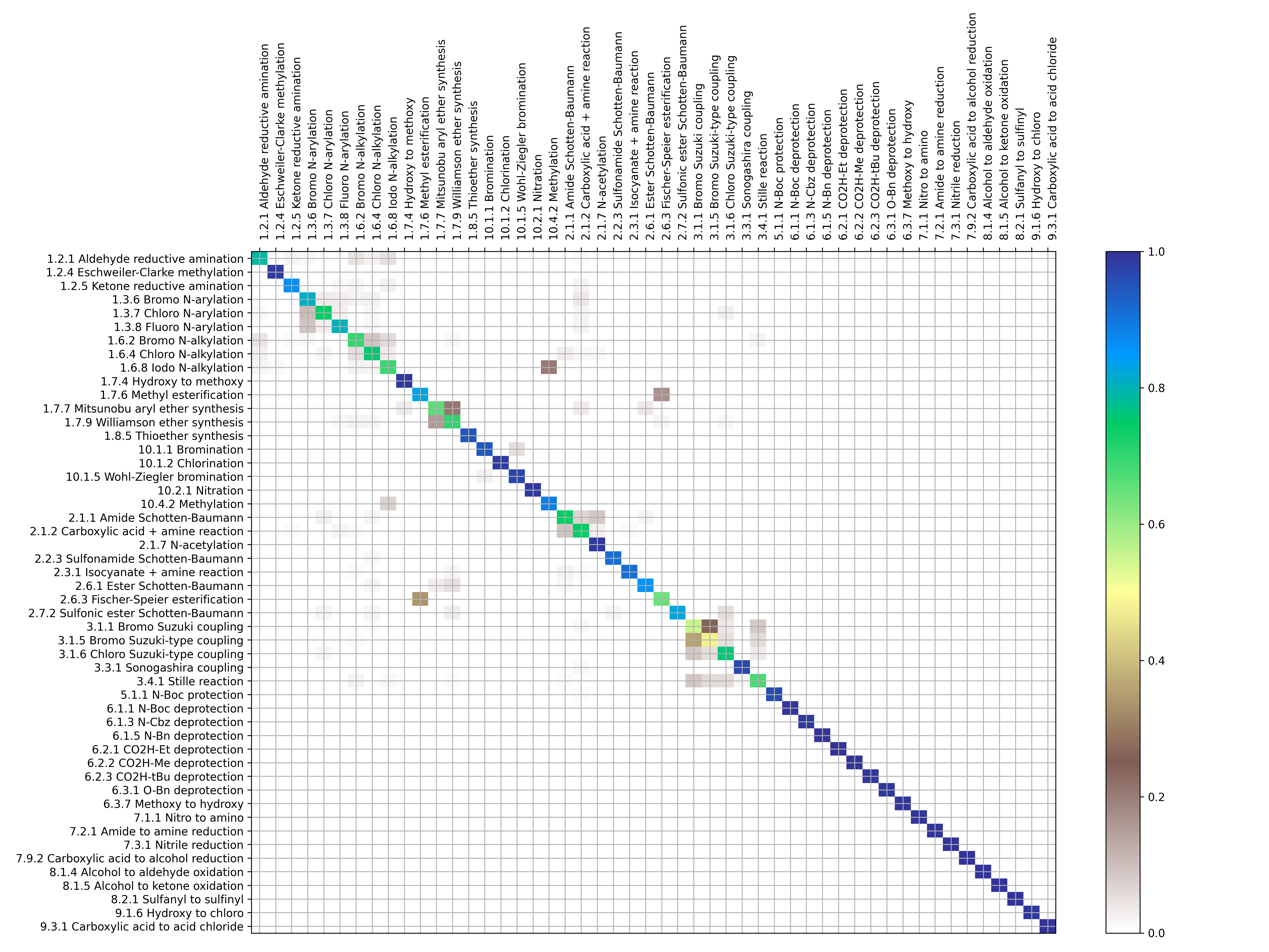
**

**Figure S8.** Confusion matrix for *rxnfp* (10k).


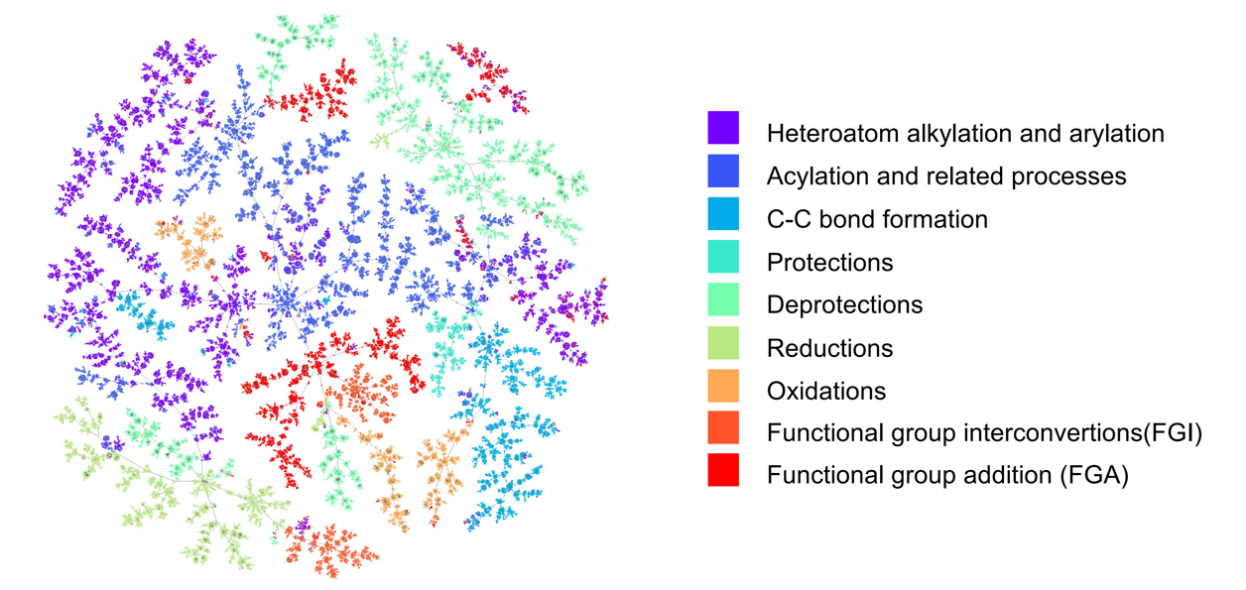


**Figure S9.** TMAP2 of the Schneider 50k dataset. Using the embeddings of CACLENS-EU as reaction fingerprints to analyze the reaction properties

**
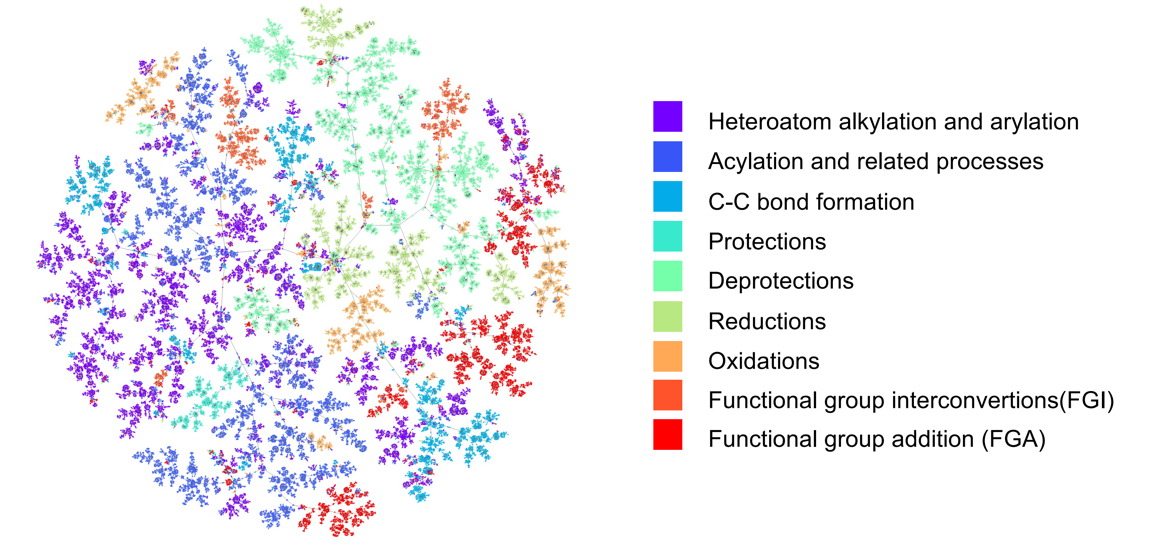
**

**Figure S10.** TMAP2 of the Schneider 50k dataset. Using the embeddings of CACLENS-EF as reaction fingerprints to analyze the reaction properties


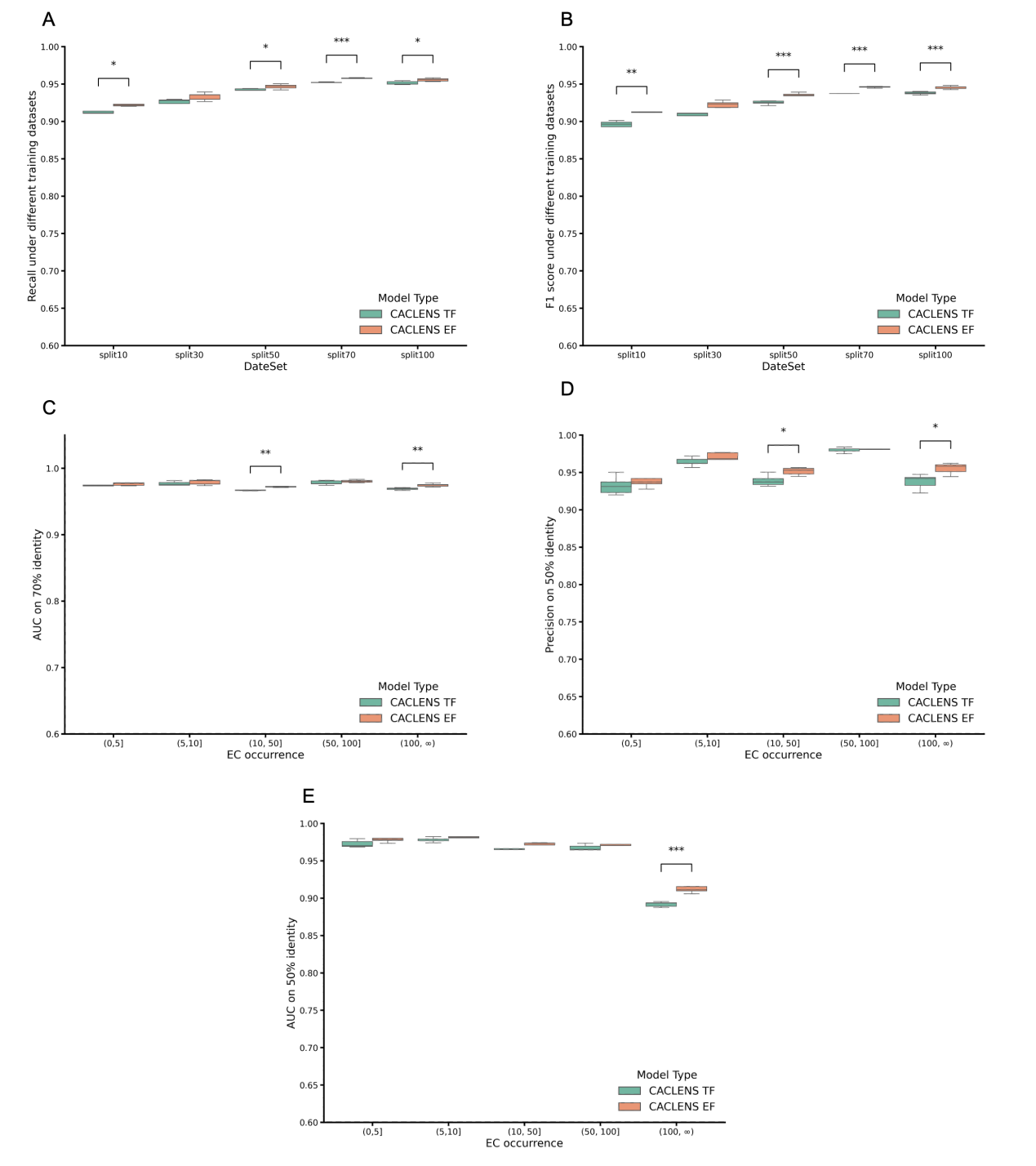


**Figure S11.** Performance of EC number prediction with five-fold cross-validation.

**A-B.** Results of recall (A) and F1 Score (B) for CACLENS-EF and CACLENS-TF on five datasets.

**C.** The ROC-AUC binned plot of five-fold cross-validation for CACLENS-EF and CACLENS-TF using the test set with less than 70% identity to the training set.

**D-E.** The precision (D) and ROC-AUC (E) binned plot of five-fold cross-validation for our two models using the test set with less than 50% identity to the training set.


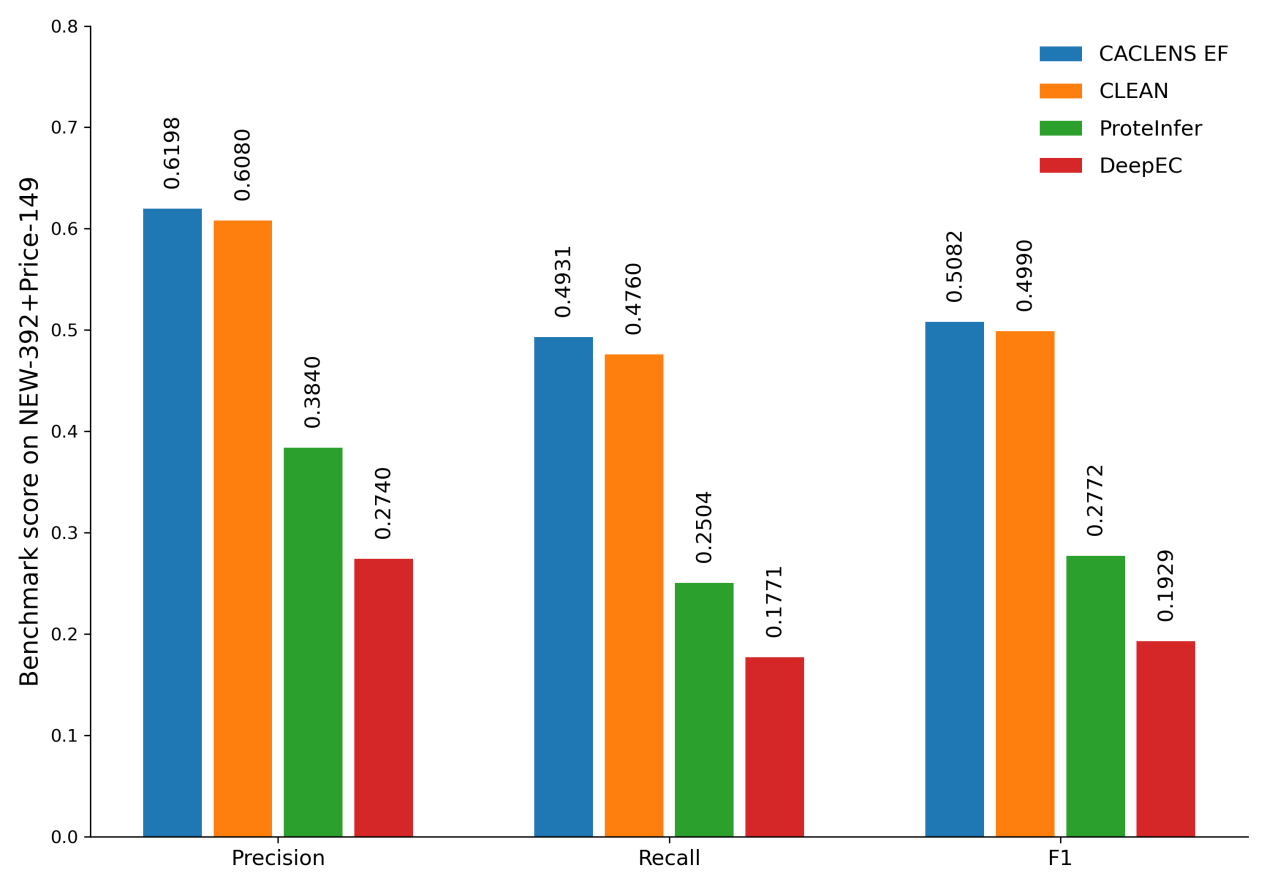


**Figure S12.** Evaluation of the performance in the New-392+Price-149 dataset.


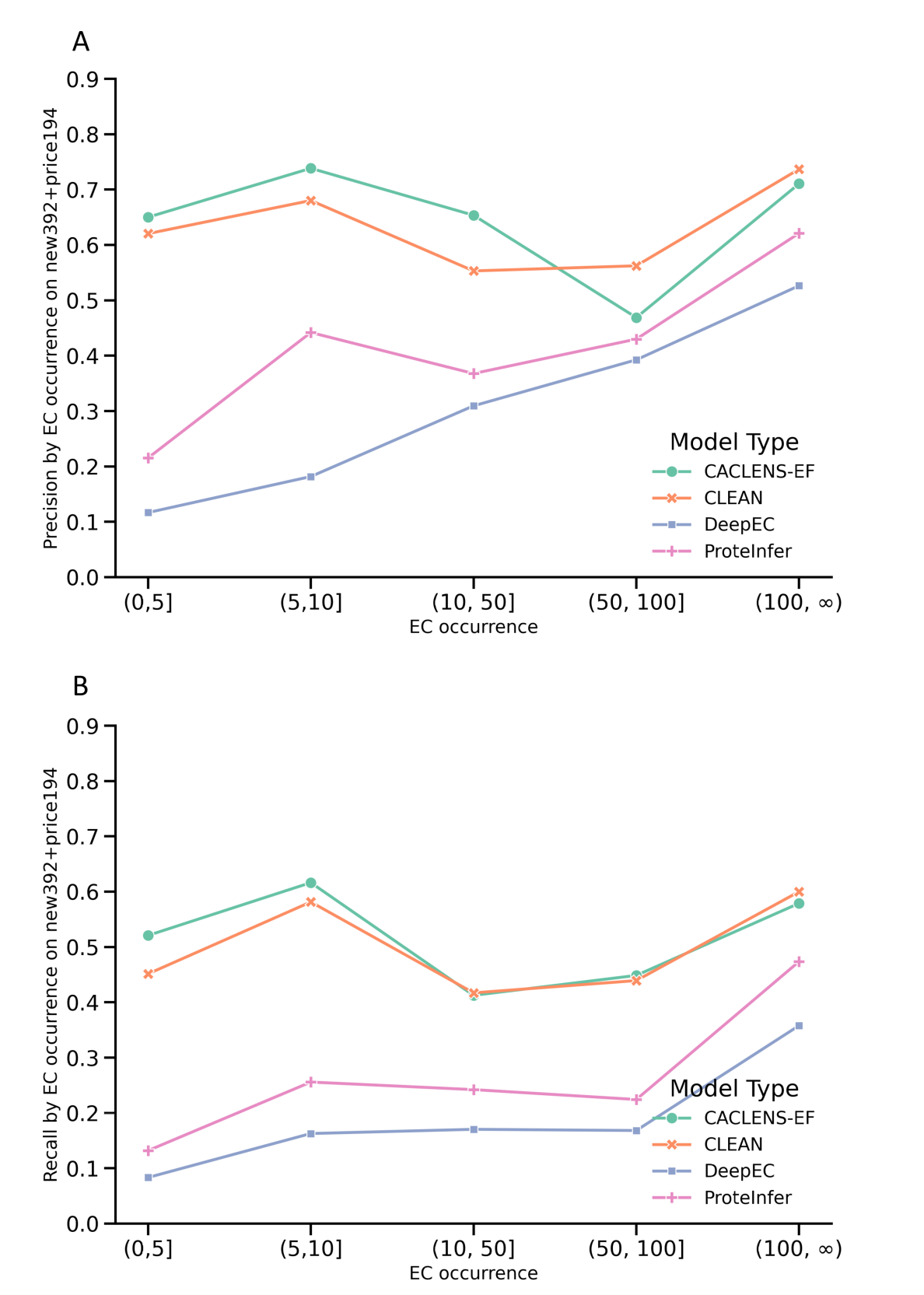


**Figure S13.** The precision(A) and recall(B) binned plot for CACLENS-EF. The bins are organized based on the frequency with which the EC number appears in the training set.


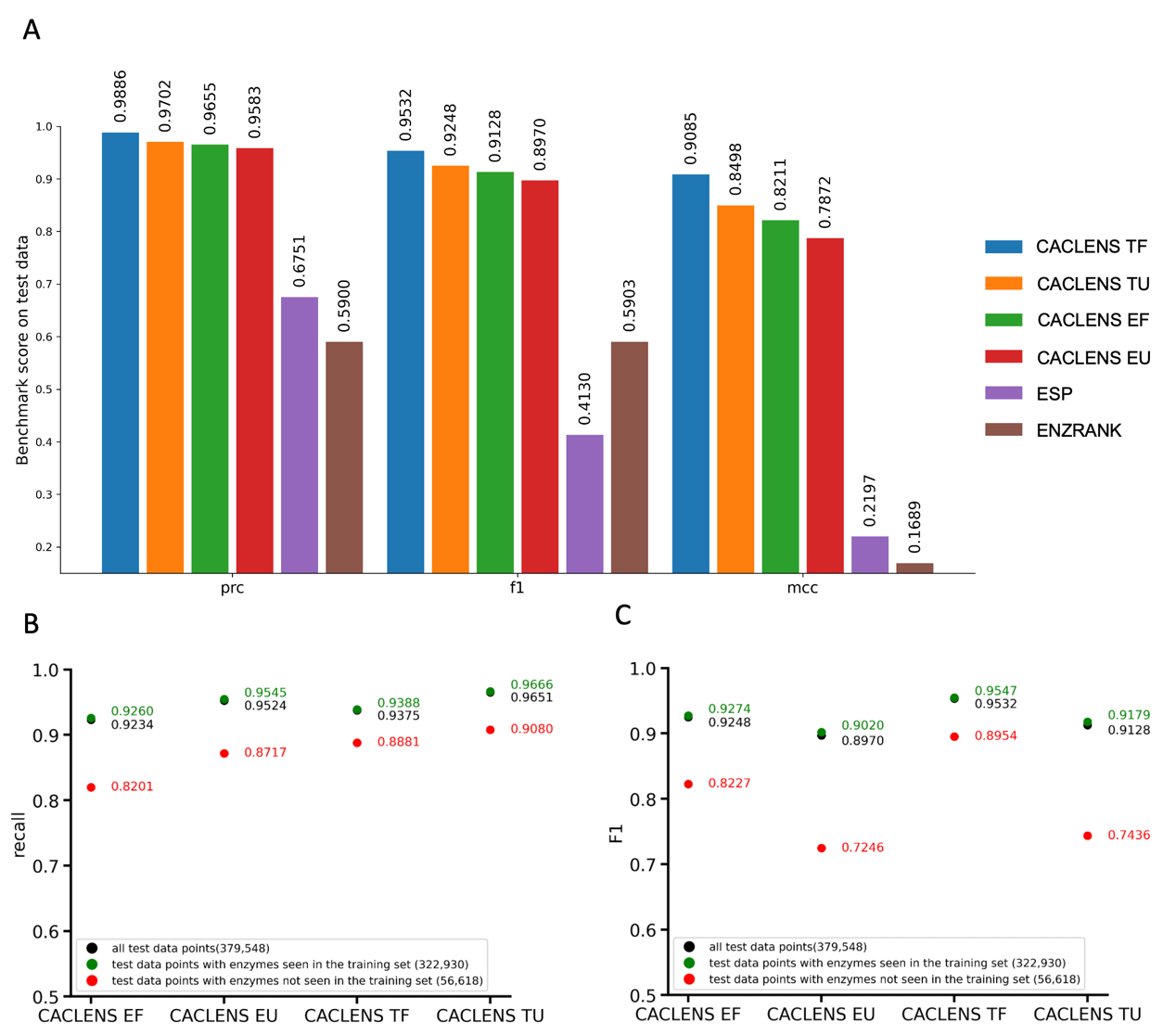


**Figure S14.** Benchmarking CACLENS on Reaction Feasibility Prediction

**A. Benchmark score of reaction prediction.** Performance in terms of PRC-AUC, F1 score and MCC parameters, tested on our test set, comparing CACLENS (CACLENS-TF, TU, EF, EU) with the ESP and ENZRANK.

**B-C.** **The performance of CACLENS in terms of Recall(B) and F1 score (C).**

**Fig S15.** Degradation rates of ZD7 on ZEN and α-ZOL in mycotoxin contaminated wheat flour.

Mycotoxin standards were added to wheat flour at a final concentration of 1000 ng/g. 1 ug of purified enzyme ZD7 and 1 mL water were mixed with 1 g mycotoxin-contaminated wheat flour. After incubation in the same condition as used in buffer, the mycotoxins were extracted and quantified using LC−MS.

**
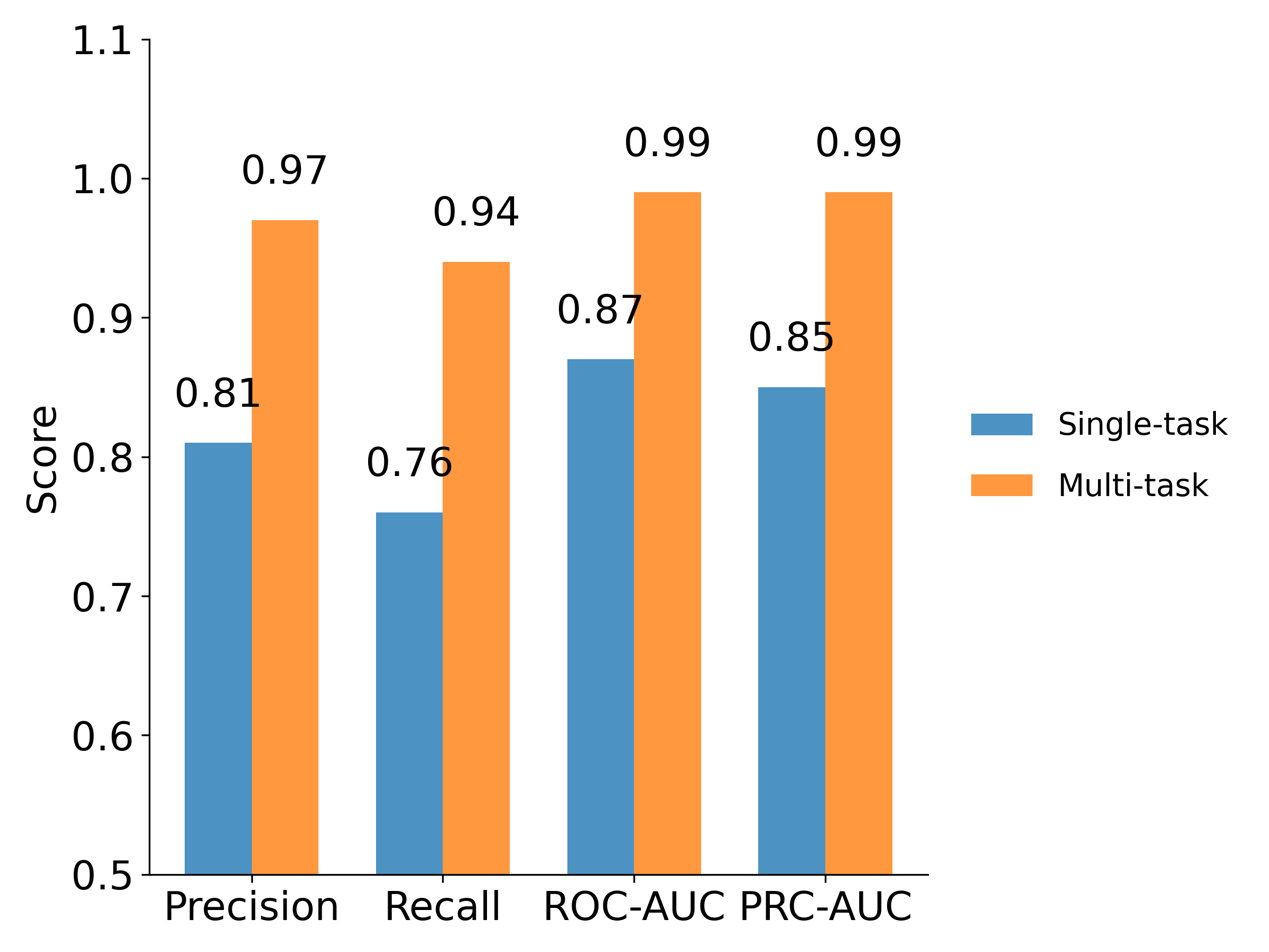
**

**Figure S16.** Single-task vs Multi-task Architecture in Reaction Feasibility Prediction

**
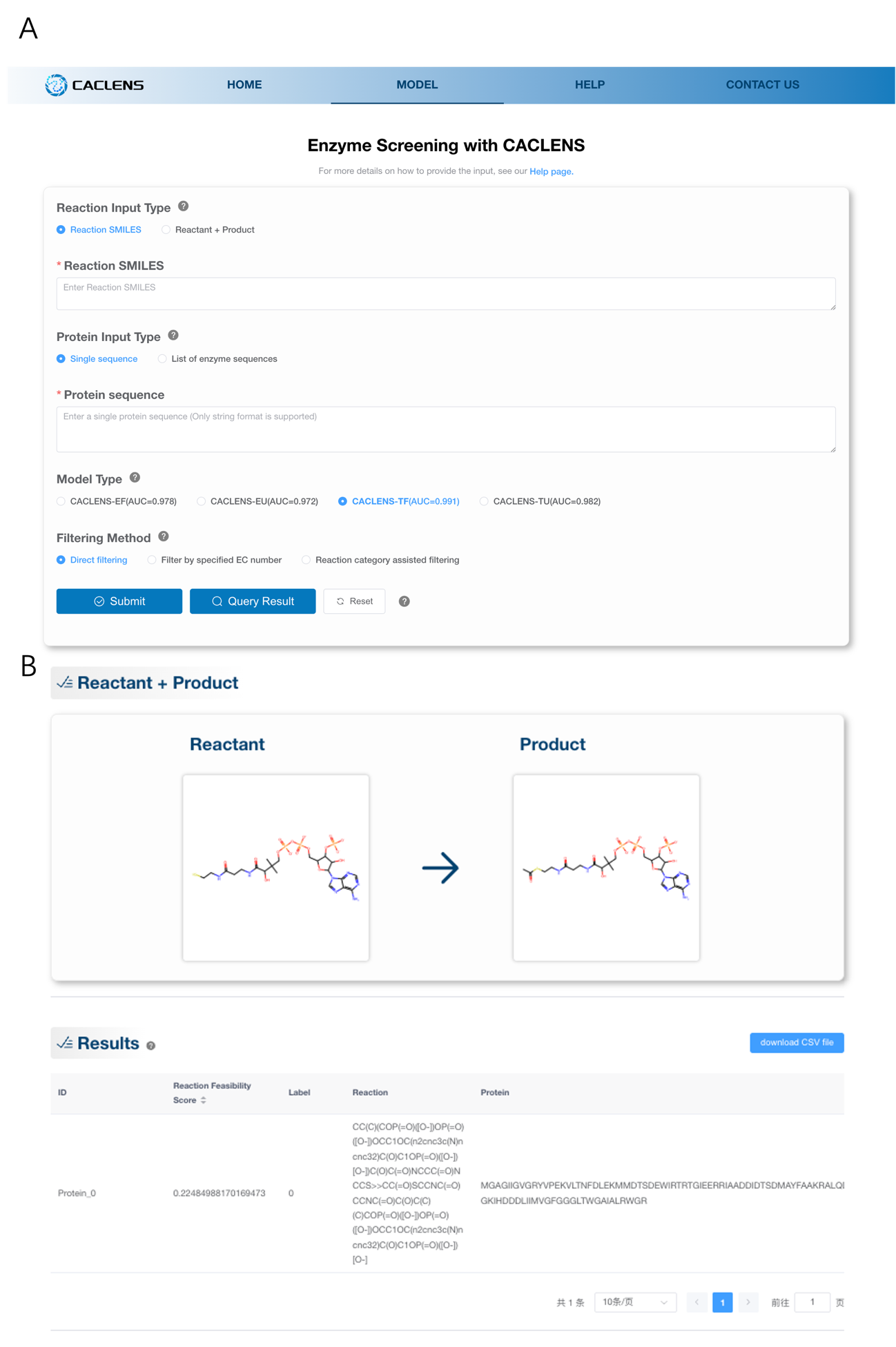
**

**Figure S17.** CACLENS Web Server

1. **Model Page** **of Web Server**
2. **Results Page of Web Server**
3. **Supplementary Table**

**Table S1.** The random classification performance on the Schneider 50k dataset. (Using logistic regression for reaction type prediction.)

| **Index** | **Recall** | **Precision** | **F1-Score** | **Class Name** | **Class** |
| --- | --- | --- | --- | --- | --- |
| 0 | 0.0195 | 0.0139 | 0.0162 | Aldehyde reductive amination | 1.2.1 |
| 1 | 0.0471 | 0.0559 | 0.0511 | Eschweiler-Clarke methylation | 1.2.4 |
| 2 | 0.0439 | 0.0507 | 0.0471 | Ketone reductive amination | 1.2.5 |
| 3 | 0.0144 | 0.0148 | 0.0146 | Bromo N-arylation | 1.3.6 |
| 4 | 0.0255 | 0.017 | 0.0204 | Chloro N-arylation | 1.3.7 |
| 5 | 0.0406 | 0.0179 | 0.0248 | Fluoro N-arylation | 1.3.8 |
| 6 | 0.0515 | 0.0263 | 0.0348 | Bromo N-alkylation | 1.6.2 |
| 7 | 0.0078 | 0.0051 | 0.0062 | Chloro N-alkylation | 1.6.4 |
| 8 | 0.0066 | 0.007 | 0.0068 | Iodo N-alkylation | 1.6.8 |
| 9 | 0.0208 | 0.0223 | 0.0215 | Hydroxy to methoxy | 1.7.4 |
| 10 | 0.0015 | 0.0018 | 0.0016 | Methyl esterification | 1.7.6 |
| 11 | 0.0134 | 0.0143 | 0.0138 | Mitsunobu aryl ether synthesis | 1.7.7 |
| 12 | 0.0304 | 0.0253 | 0.0276 | Williamson ether synthesis | 1.7.9 |
| 13 | 0.007 | 0.0111 | 0.0086 | Thioether synthesis | 1.8.5 |
| 14 | 0.0128 | 0.0203 | 0.0157 | Bromination | 10.1.1 |
| 15 | 0.0339 | 0.0279 | 0.0306 | Chlorination | 10.1.2 |
| 16 | 0.0126 | 0.0115 | 0.012 | Wohl-Ziegler bromination | 10.1.5 |
| 17 | 0.0088 | 0.0123 | 0.0102 | Nitration | 10.2.1 |
| 18 | 0.0223 | 0.0165 | 0.019 | Methylation | 10.4.2 |
| 19 | 0.0066 | 0.0069 | 0.0067 | Amide Schotten-Baumann | 2.1.1 |
| 20 | 0.0646 | 0.031 | 0.0419 | Carboxylic acid + amine reaction | 2.1.2 |
| 21 | 0.023 | 0.0237 | 0.0234 | N-acetylation | 2.1.7 |
| 22 | 0.0008 | 0.0017 | 0.0011 | Sulfonamide Schotten-Baumann | 2.2.3 |
| 23 | 0.0224 | 0.0298 | 0.0256 | Isocyanate + amine reaction | 2.3.1 |
| 24 | 0.0179 | 0.0185 | 0.0182 | Ester Schotten-Baumann | 2.6.1 |
| 25 | 0.0215 | 0.0202 | 0.0208 | Fischer-Speier esterification | 2.6.3 |
| 26 | 0.0084 | 0.0126 | 0.0101 | Sulfonic ester Schotten-Baumann | 2.7.2 |
| 27 | 0.0047 | 0.0025 | 0.0032 | Bromo Suzuki coupling | 3.1.1 |
| 28 | 0.017 | 0.0167 | 0.0169 | Bromo Suzuki-type coupling | 3.1.5 |
| 29 | 0.0016 | 0.0014 | 0.0015 | Chloro Suzuki-type coupling | 3.1.6 |
| 30 | 0.001 | 0.0013 | 0.0012 | Sonogashira coupling | 3.3.1 |
| 31 | 0.0502 | 0.037 | 0.0426 | Stille reaction | 3.4.1 |
| 32 | 0.0103 | 0.0111 | 0.0107 | N-Boc protection | 5.1.1 |
| 33 | 0.0681 | 0.0644 | 0.0662 | N-Boc deprotection | 6.1.1 |
| 34 | 0.008 | 0.0102 | 0.009 | N-Cbz deprotection | 6.1.3 |
| 35 | 0.0292 | 0.0275 | 0.0283 | N-Bn deprotection | 6.1.5 |
| 36 | 0.0164 | 0.0136 | 0.0149 | CO2H-Et deprotection | 6.2.1 |
| 37 | 0.0088 | 0.0092 | 0.009 | CO2H-Me deprotection | 6.2.2 |
| 38 | 0.0088 | 0.0089 | 0.0089 | CO2H-tBu deprotection | 6.2.3 |
| 39 | 0.0175 | 0.0188 | 0.0181 | O-Bn deprotection | 6.3.1 |
| 40 | 0.0291 | 0.0276 | 0.0283 | Methoxy to hydroxy | 6.3.7 |
| 41 | 0 | 0 | 0 | Nitro to amino | 7.1.1 |
| 42 | 0.0101 | 0.0108 | 0.0104 | Amide to amine reduction | 7.2.1 |
| 43 | 0.0038 | 0.0053 | 0.0044 | Nitrile reduction | 7.3.1 |
| 44 | 0.0203 | 0.0208 | 0.0206 | Carboxylic acid to alcohol reduction | 7.9.2 |
| 45 | 0.0892 | 0.0838 | 0.0864 | Alcohol to aldehyde oxidation | 8.1.4 |
| 46 | 0.0281 | 0.0335 | 0.0306 | Alcohol to ketone oxidation | 8.1.5 |
| 47 | 0.0088 | 0.0091 | 0.0089 | Sulfanyl to sulfinyl | 8.2.1 |
| 48 | 0.0063 | 0.0081 | 0.0071 | Hydroxy to chloro | 9.1.6 |
| 49 | 0.0013 | 0.0013 | 0.0013 | Carboxylic acid to acid chloride | 9.3.1 |

**Table S2.** The CACLENS-EU's classification performance on the Schneider 50k dataset. Treat the encoding of Schneider 50k by CACLENS-EU as reaction fingerprints and use logistic regression for reaction type prediction.

| **Index** | **Recall** | **Precision** | **F1-Score** | **Class Name** | **Class** |
| --- | --- | --- | --- | --- | --- |
| 0 | 0.7371 | 0.6608 | 0.6969 | Aldehyde reductive amination | 1.2.1 |
| 1 | 0.7712 | 0.7753 | 0.7733 | Eschweiler-Clarke methylation | 1.2.4 |
| 2 | 0.761 | 0.9334 | 0.8384 | Ketone reductive amination | 1.2.5 |
| 3 | 0.7023 | 0.6453 | 0.6726 | Bromo N-arylation | 1.3.6 |
| 4 | 0.7687 | 0.8828 | 0.8218 | Chloro N-arylation | 1.3.7 |
| 5 | 0.8677 | 0.8396 | 0.8534 | Fluoro N-arylation | 1.3.8 |
| 6 | 0.6649 | 0.6192 | 0.6413 | Bromo N-alkylation | 1.6.2 |
| 7 | 0.6571 | 0.566 | 0.6082 | Chloro N-alkylation | 1.6.4 |
| 8 | 0.6053 | 0.5732 | 0.5888 | Iodo N-alkylation | 1.6.8 |
| 9 | 0.9052 | 0.8658 | 0.8851 | Hydroxy to methoxy | 1.7.4 |
| 10 | 0.6956 | 0.7356 | 0.715 | Methyl esterification | 1.7.6 |
| 11 | 0.7693 | 0.7282 | 0.7482 | Mitsunobu aryl ether synthesis | 1.7.7 |
| 12 | 0.6661 | 0.5359 | 0.5939 | Williamson ether synthesis | 1.7.9 |
| 13 | 0.8664 | 0.9456 | 0.9043 | Thioether synthesis | 1.8.5 |
| 14 | 0.9196 | 0.9315 | 0.9255 | Bromination | 10.1.1 |
| 15 | 0.9323 | 0.9496 | 0.9409 | Chlorination | 10.1.2 |
| 16 | 0.9534 | 0.9474 | 0.9504 | Wohl-Ziegler bromination | 10.1.5 |
| 17 | 0.9912 | 0.99 | 0.9906 | Nitration | 10.2.1 |
| 18 | 0.795 | 0.659 | 0.7206 | Methylation | 10.4.2 |
| 19 | 0.7743 | 0.6413 | 0.7015 | Amide Schotten-Baumann | 2.1.1 |
| 20 | 0.6646 | 0.7367 | 0.6988 | Carboxylic acid + amine reaction | 2.1.2 |
| 21 | 0.8986 | 0.8548 | 0.8762 | N-acetylation | 2.1.7 |
| 22 | 0.8288 | 0.9575 | 0.8885 | Sulfonamide Schotten-Baumann | 2.2.3 |
| 23 | 0.8796 | 0.9584 | 0.9173 | Isocyanate + amine reaction | 2.3.1 |
| 24 | 0.8483 | 0.8156 | 0.8316 | Ester Schotten-Baumann | 2.6.1 |
| 25 | 0.7204 | 0.7171 | 0.7188 | Fischer-Speier esterification | 2.6.3 |
| 26 | 0.8768 | 0.9448 | 0.9095 | Sulfonic ester Schotten-Baumann | 2.7.2 |
| 27 | 0.6285 | 0.5075 | 0.5616 | Bromo Suzuki coupling | 3.1.1 |
| 28 | 0.5997 | 0.6579 | 0.6275 | Bromo Suzuki-type coupling | 3.1.5 |
| 29 | 0.8306 | 0.8333 | 0.832 | Chloro Suzuki-type coupling | 3.1.6 |
| 30 | 0.8658 | 0.9711 | 0.9154 | Sonogashira coupling | 3.3.1 |
| 31 | 0.6442 | 0.7352 | 0.6867 | Stille reaction | 3.4.1 |
| 32 | 0.9381 | 0.9357 | 0.9369 | N-Boc protection | 5.1.1 |
| 33 | 0.995 | 0.9912 | 0.9931 | N-Boc deprotection | 6.1.1 |
| 34 | 0.9786 | 0.9946 | 0.9865 | N-Cbz deprotection | 6.1.3 |
| 35 | 0.9924 | 0.9751 | 0.9837 | N-Bn deprotection | 6.1.5 |
| 36 | 0.9596 | 0.8609 | 0.9076 | CO2H-Et deprotection | 6.2.1 |
| 37 | 0.8535 | 0.9185 | 0.8848 | CO2H-Me deprotection | 6.2.2 |
| 38 | 0.9787 | 0.9962 | 0.9873 | CO2H-tBu deprotection | 6.2.3 |
| 39 | 0.9749 | 0.9836 | 0.9792 | O-Bn deprotection | 6.3.1 |
| 40 | 0.9482 | 0.9494 | 0.9488 | Methoxy to hydroxy | 6.3.7 |
| 41 | 0.9875 | 0.995 | 0.9912 | Nitro to amino | 7.1.1 |
| 42 | 0.9886 | 0.9714 | 0.9799 | Amide to amine reduction | 7.2.1 |
| 43 | 0.9937 | 0.9789 | 0.9862 | Nitrile reduction | 7.3.1 |
| 44 | 0.9873 | 0.9873 | 0.9873 | Carboxylic acid to alcohol reduction | 7.9.2 |
| 45 | 0.9359 | 0.9588 | 0.9472 | Alcohol to aldehyde oxidation | 8.1.4 |
| 46 | 0.945 | 0.9261 | 0.9354 | Alcohol to ketone oxidation | 8.1.5 |
| 47 | 0.995 | 0.9684 | 0.9815 | Sulfanyl to sulfinyl | 8.2.1 |
| 48 | 0.9723 | 0.9923 | 0.9822 | Hydroxy to chloro | 9.1.6 |
| 49 | 0.995 | 0.9863 | 0.9906 | Carboxylic acid to acid chloride | 9.3.1 |

**Table S3.** The CACLENS-EF's classification performance on the Schneider 50k dataset. Treat the encoding of Schneider 50k by CACLENS-EF as reaction fingerprints and use logistic regression for reaction type prediction.

| **Index** | **Recall** | **Precision** | **F1-Score** | **Class Name** | **Class** |
| --- | --- | --- | --- | --- | --- |
| 0 | 0.9325 | 0.9409 | 0.9367 | Aldehyde reductive amination | 1.2.1 |
| 1 | 0.8954 | 0.8384 | 0.866 | Eschweiler-Clarke methylation | 1.2.4 |
| 2 | 0.978 | 0.9631 | 0.9705 | Ketone reductive amination | 1.2.5 |
| 3 | 0.9651 | 0.9533 | 0.9592 | Bromo N-arylation | 1.3.6 |
| 4 | 0.9609 | 0.9826 | 0.9716 | Chloro N-arylation | 1.3.7 |
| 5 | 0.9753 | 0.9567 | 0.9659 | Fluoro N-arylation | 1.3.8 |
| 6 | 0.866 | 0.8827 | 0.8742 | Bromo N-alkylation | 1.6.2 |
| 7 | 0.826 | 0.9191 | 0.87 | Chloro N-alkylation | 1.6.4 |
| 8 | 0.7056 | 0.7886 | 0.7448 | Iodo N-alkylation | 1.6.8 |
| 9 | 0.913 | 0.9166 | 0.9148 | Hydroxy to methoxy | 1.7.4 |
| 10 | 0.7838 | 0.768 | 0.7758 | Methyl esterification | 1.7.6 |
| 11 | 0.9315 | 0.9371 | 0.9343 | Mitsunobu aryl ether synthesis | 1.7.7 |
| 12 | 0.8232 | 0.8715 | 0.8466 | Williamson ether synthesis | 1.7.9 |
| 13 | 0.9831 | 0.9667 | 0.9748 | Thioether synthesis | 1.8.5 |
| 14 | 0.9311 | 0.9456 | 0.9383 | Bromination | 10.1.1 |
| 15 | 0.9531 | 0.9606 | 0.9569 | Chlorination | 10.1.2 |
| 16 | 0.9584 | 0.9147 | 0.936 | Wohl-Ziegler bromination | 10.1.5 |
| 17 | 0.9962 | 0.9925 | 0.9944 | Nitration | 10.2.1 |
| 18 | 0.7015 | 0.7727 | 0.7354 | Methylation | 10.4.2 |
| 19 | 0.9186 | 0.9485 | 0.9333 | Amide Schotten-Baumann | 2.1.1 |
| 20 | 0.9625 | 0.9625 | 0.9625 | Carboxylic acid + amine reaction | 2.1.2 |
| 21 | 0.9405 | 0.8935 | 0.9164 | N-acetylation | 2.1.7 |
| 22 | 0.9992 | 0.9946 | 0.9969 | Sulfonamide Schotten-Baumann | 2.2.3 |
| 23 | 0.9958 | 0.9983 | 0.9971 | Isocyanate + amine reaction | 2.3.1 |
| 24 | 0.9842 | 0.977 | 0.9806 | Ester Schotten-Baumann | 2.6.1 |
| 25 | 0.7465 | 0.7606 | 0.7535 | Fischer-Speier esterification | 2.6.3 |
| 26 | 0.9992 | 0.9962 | 0.9977 | Sulfonic ester Schotten-Baumann | 2.7.2 |
| 27 | 0.9322 | 0.8012 | 0.8618 | Bromo Suzuki coupling | 3.1.1 |
| 28 | 0.9114 | 0.937 | 0.924 | Bromo Suzuki-type coupling | 3.1.5 |
| 29 | 0.9613 | 0.946 | 0.9536 | Chloro Suzuki-type coupling | 3.1.6 |
| 30 | 0.9979 | 0.9827 | 0.9903 | Sonogashira coupling | 3.3.1 |
| 31 | 0.8009 | 0.8841 | 0.8405 | Stille reaction | 3.4.1 |
| 32 | 0.991 | 0.9884 | 0.9897 | N-Boc protection | 5.1.1 |
| 33 | 0.995 | 0.9975 | 0.9962 | N-Boc deprotection | 6.1.1 |
| 34 | 0.9906 | 0.9946 | 0.9926 | N-Cbz deprotection | 6.1.3 |
| 35 | 0.9911 | 0.9924 | 0.9918 | N-Bn deprotection | 6.1.5 |
| 36 | 0.9672 | 0.9709 | 0.969 | CO2H-Et deprotection | 6.2.1 |
| 37 | 0.9735 | 0.9519 | 0.9625 | CO2H-Me deprotection | 6.2.2 |
| 38 | 0.9962 | 0.9962 | 0.9962 | CO2H-tBu deprotection | 6.2.3 |
| 39 | 0.9862 | 0.9912 | 0.9887 | O-Bn deprotection | 6.3.1 |
| 40 | 0.9735 | 0.9821 | 0.9778 | Methoxy to hydroxy | 6.3.7 |
| 41 | 0.9925 | 0.9937 | 0.9931 | Nitro to amino | 7.1.1 |
| 42 | 0.9912 | 0.9825 | 0.9868 | Amide to amine reduction | 7.2.1 |
| 43 | 1 | 0.9925 | 0.9962 | Nitrile reduction | 7.3.1 |
| 44 | 0.9911 | 0.9874 | 0.9892 | Carboxylic acid to alcohol reduction | 7.9.2 |
| 45 | 0.9837 | 0.9775 | 0.9806 | Alcohol to aldehyde oxidation | 8.1.4 |
| 46 | 0.991 | 0.9798 | 0.9854 | Alcohol to ketone oxidation | 8.1.5 |
| 47 | 0.9975 | 0.9661 | 0.9815 | Sulfanyl to sulfinyl | 8.2.1 |
| 48 | 0.9849 | 0.9787 | 0.9818 | Hydroxy to chloro | 9.1.6 |
| 49 | 0.9975 | 0.9913 | 0.9944 | Carboxylic acid to acid chloride | 9.3.1 |

**Table S4.** The CACLENS-EU's classification performance on the Schneider 50k dataset. Treat the encoding of Schneider 50k by CACLENS-EU as reaction fingerprints and perform reaction type prediction through Euclidean distance search.

| **Index** | **Recall** | **Precision** | **F1-Score** | **Class Name** | **Class** |
| --- | --- | --- | --- | --- | --- |
| 0 | 0.7265 | 0.6534 | 0.688 | Aldehyde reductive amination | 1.2.1 |
| 1 | 0.7425 | 0.7655 | 0.7538 | Eschweiler-Clarke methylation | 1.2.4 |
| 2 | 0.7907 | 0.9121 | 0.8471 | Ketone reductive amination | 1.2.5 |
| 3 | 0.6858 | 0.6816 | 0.6837 | Bromo N-arylation | 1.3.6 |
| 4 | 0.7551 | 0.8315 | 0.7914 | Chloro N-arylation | 1.3.7 |
| 5 | 0.8466 | 0.8743 | 0.8602 | Fluoro N-arylation | 1.3.8 |
| 6 | 0.7113 | 0.5766 | 0.6369 | Bromo N-alkylation | 1.6.2 |
| 7 | 0.6571 | 0.5464 | 0.5967 | Chloro N-alkylation | 1.6.4 |
| 8 | 0.6053 | 0.5697 | 0.5869 | Iodo N-alkylation | 1.6.8 |
| 9 | 0.9104 | 0.8829 | 0.8964 | Hydroxy to methoxy | 1.7.4 |
| 10 | 0.6912 | 0.7367 | 0.7132 | Methyl esterification | 1.7.6 |
| 11 | 0.7798 | 0.7034 | 0.7396 | Mitsunobu aryl ether synthesis | 1.7.7 |
| 12 | 0.6214 | 0.5225 | 0.5677 | Williamson ether synthesis | 1.7.9 |
| 13 | 0.8584 | 0.9451 | 0.8997 | Thioether synthesis | 1.8.5 |
| 14 | 0.9209 | 0.9105 | 0.9157 | Bromination | 10.1.1 |
| 15 | 0.9375 | 0.9511 | 0.9443 | Chlorination | 10.1.2 |
| 16 | 0.9395 | 0.954 | 0.9467 | Wohl-Ziegler bromination | 10.1.5 |
| 17 | 0.9938 | 0.9975 | 0.9956 | Nitration | 10.2.1 |
| 18 | 0.7852 | 0.6471 | 0.7095 | Methylation | 10.4.2 |
| 19 | 0.7756 | 0.6124 | 0.6844 | Amide Schotten-Baumann | 2.1.1 |
| 20 | 0.65 | 0.7256 | 0.6857 | Carboxylic acid + amine reaction | 2.1.2 |
| 21 | 0.8743 | 0.8627 | 0.8685 | N-acetylation | 2.1.7 |
| 22 | 0.8116 | 0.9683 | 0.883 | Sulfonamide Schotten-Baumann | 2.2.3 |
| 23 | 0.8547 | 0.9662 | 0.907 | Isocyanate + amine reaction | 2.3.1 |
| 24 | 0.8504 | 0.7873 | 0.8176 | Ester Schotten-Baumann | 2.6.1 |
| 25 | 0.722 | 0.6994 | 0.7105 | Fischer-Speier esterification | 2.6.3 |
| 26 | 0.8577 | 0.9436 | 0.8986 | Sulfonic ester Schotten-Baumann | 2.7.2 |
| 27 | 0.6308 | 0.5009 | 0.5584 | Bromo Suzuki coupling | 3.1.1 |
| 28 | 0.6235 | 0.6332 | 0.6283 | Bromo Suzuki-type coupling | 3.1.5 |
| 29 | 0.8242 | 0.878 | 0.8502 | Chloro Suzuki-type coupling | 3.1.6 |
| 30 | 0.8369 | 0.983 | 0.9041 | Sonogashira coupling | 3.3.1 |
| 31 | 0.6959 | 0.7161 | 0.7059 | Stille reaction | 3.4.1 |
| 32 | 0.9317 | 0.9718 | 0.9513 | N-Boc protection | 5.1.1 |
| 33 | 0.9887 | 0.9949 | 0.9918 | N-Boc deprotection | 6.1.1 |
| 34 | 0.9813 | 0.9892 | 0.9852 | N-Cbz deprotection | 6.1.3 |
| 35 | 0.9899 | 0.9861 | 0.988 | N-Bn deprotection | 6.1.5 |
| 36 | 0.9584 | 0.8686 | 0.9113 | CO2H-Et deprotection | 6.2.1 |
| 37 | 0.8763 | 0.906 | 0.8909 | CO2H-Me deprotection | 6.2.2 |
| 38 | 0.9762 | 0.9949 | 0.9854 | CO2H-tBu deprotection | 6.2.3 |
| 39 | 0.9712 | 0.9835 | 0.9773 | O-Bn deprotection | 6.3.1 |
| 40 | 0.9406 | 0.9625 | 0.9514 | Methoxy to hydroxy | 6.3.7 |
| 41 | 0.9875 | 0.9962 | 0.9918 | Nitro to amino | 7.1.1 |
| 42 | 0.9874 | 0.9849 | 0.9861 | Amide to amine reduction | 7.2.1 |
| 43 | 0.9912 | 0.9862 | 0.9887 | Nitrile reduction | 7.3.1 |
| 44 | 0.9886 | 0.9886 | 0.9886 | Carboxylic acid to alcohol reduction | 7.9.2 |
| 45 | 0.9435 | 0.9579 | 0.9506 | Alcohol to aldehyde oxidation | 8.1.4 |
| 46 | 0.9463 | 0.9332 | 0.9397 | Alcohol to ketone oxidation | 8.1.5 |
| 47 | 0.9925 | 0.9718 | 0.9821 | Sulfanyl to sulfinyl | 8.2.1 |
| 48 | 0.976 | 0.991 | 0.9835 | Hydroxy to chloro | 9.1.6 |
| 49 | 0.9912 | 0.9888 | 0.99 | Carboxylic acid to acid chloride | 9.3.1 |

**Table S5.** The CACLENS-EF's classification performance on the Schneider 50k dataset. Treat the encoding of Schneider 50k by CACLENS-EF as reaction fingerprints and perform reaction type prediction through Euclidean distance search.

| **Index** | **Recall** | **Precision** | **F1-Score** | **Class Name** | **Class** |
| --- | --- | --- | --- | --- | --- |
| 0 | 0.8917 | 0.9211 | 0.9061 | Aldehyde reductive amination | 1.2.1 |
| 1 | 0.8706 | 0.8484 | 0.8594 | Eschweiler-Clarke methylation | 1.2.4 |
| 2 | 0.9496 | 0.9496 | 0.9496 | Ketone reductive amination | 1.2.5 |
| 3 | 0.9199 | 0.918 | 0.919 | Bromo N-arylation | 1.3.6 |
| 4 | 0.9405 | 0.9201 | 0.9302 | Chloro N-arylation | 1.3.7 |
| 5 | 0.9541 | 0.9092 | 0.9312 | Fluoro N-arylation | 1.3.8 |
| 6 | 0.854 | 0.8174 | 0.8353 | Bromo N-alkylation | 1.6.2 |
| 7 | 0.8052 | 0.801 | 0.8031 | Chloro N-alkylation | 1.6.4 |
| 8 | 0.6957 | 0.7085 | 0.7021 | Iodo N-alkylation | 1.6.8 |
| 9 | 0.8844 | 0.9166 | 0.9002 | Hydroxy to methoxy | 1.7.4 |
| 10 | 0.7132 | 0.745 | 0.7288 | Methyl esterification | 1.7.6 |
| 11 | 0.8899 | 0.8654 | 0.8775 | Mitsunobu aryl ether synthesis | 1.7.7 |
| 12 | 0.8107 | 0.7774 | 0.7937 | Williamson ether synthesis | 1.7.9 |
| 13 | 0.9531 | 0.9503 | 0.9517 | Thioether synthesis | 1.8.5 |
| 14 | 0.9337 | 0.9385 | 0.9361 | Bromination | 10.1.1 |
| 15 | 0.9518 | 0.9556 | 0.9537 | Chlorination | 10.1.2 |
| 16 | 0.9521 | 0.922 | 0.9368 | Wohl-Ziegler bromination | 10.1.5 |
| 17 | 0.9888 | 0.9912 | 0.99 | Nitration | 10.2.1 |
| 18 | 0.6736 | 0.7385 | 0.7046 | Methylation | 10.4.2 |
| 19 | 0.9003 | 0.9122 | 0.9062 | Amide Schotten-Baumann | 2.1.1 |
| 20 | 0.9167 | 0.9462 | 0.9312 | Carboxylic acid + amine reaction | 2.1.2 |
| 21 | 0.9243 | 0.8953 | 0.9096 | N-acetylation | 2.1.7 |
| 22 | 0.9648 | 0.9888 | 0.9767 | Sulfonamide Schotten-Baumann | 2.2.3 |
| 23 | 0.9751 | 0.9958 | 0.9853 | Isocyanate + amine reaction | 2.3.1 |
| 24 | 0.961 | 0.9754 | 0.9682 | Ester Schotten-Baumann | 2.6.1 |
| 25 | 0.7327 | 0.7035 | 0.7178 | Fischer-Speier esterification | 2.6.3 |
| 26 | 0.9885 | 0.987 | 0.9878 | Sulfonic ester Schotten-Baumann | 2.7.2 |
| 27 | 0.8972 | 0.7442 | 0.8136 | Bromo Suzuki coupling | 3.1.1 |
| 28 | 0.879 | 0.8658 | 0.8724 | Bromo Suzuki-type coupling | 3.1.5 |
| 29 | 0.9468 | 0.9422 | 0.9445 | Chloro Suzuki-type coupling | 3.1.6 |
| 30 | 0.9288 | 0.9847 | 0.9559 | Sonogashira coupling | 3.3.1 |
| 31 | 0.7868 | 0.823 | 0.8045 | Stille reaction | 3.4.1 |
| 32 | 0.982 | 0.9858 | 0.9839 | N-Boc protection | 5.1.1 |
| 33 | 0.995 | 0.9987 | 0.9968 | N-Boc deprotection | 6.1.1 |
| 34 | 0.9906 | 0.9946 | 0.9926 | N-Cbz deprotection | 6.1.3 |
| 35 | 0.9899 | 0.9936 | 0.9917 | N-Bn deprotection | 6.1.5 |
| 36 | 0.9546 | 0.9718 | 0.9631 | CO2H-Et deprotection | 6.2.1 |
| 37 | 0.9747 | 0.9449 | 0.9596 | CO2H-Me deprotection | 6.2.2 |
| 38 | 0.9962 | 0.9987 | 0.9975 | CO2H-tBu deprotection | 6.2.3 |
| 39 | 0.9862 | 0.9912 | 0.9887 | O-Bn deprotection | 6.3.1 |
| 40 | 0.9747 | 0.9885 | 0.9815 | Methoxy to hydroxy | 6.3.7 |
| 41 | 0.9925 | 0.995 | 0.9937 | Nitro to amino | 7.1.1 |
| 42 | 0.9899 | 0.9849 | 0.9874 | Amide to amine reduction | 7.2.1 |
| 43 | 1 | 0.9937 | 0.9969 | Nitrile reduction | 7.3.1 |
| 44 | 0.9898 | 0.9924 | 0.9911 | Carboxylic acid to alcohol reduction | 7.9.2 |
| 45 | 0.9837 | 0.9861 | 0.9849 | Alcohol to aldehyde oxidation | 8.1.4 |
| 46 | 0.991 | 0.9823 | 0.9866 | Alcohol to ketone oxidation | 8.1.5 |
| 47 | 0.9962 | 0.9672 | 0.9815 | Sulfanyl to sulfinyl | 8.2.1 |
| 48 | 0.9836 | 0.9811 | 0.9824 | Hydroxy to chloro | 9.1.6 |
| 49 | 0.9975 | 0.995 | 0.9962 | Carboxylic acid to acid chloride | 9.3.1 |

**Table S6.** The *rxnfp* (pretrained)'s performance on the Schneider 50k dataset. (Using logistic regression for reaction type prediction.)

| **Index** | **Recall** | **Precision** | **F1-Score** | **Class Name** | **Class** |
| --- | --- | --- | --- | --- | --- |
| 0 | 0.6732 | 0.6113 | 0.6407 | Aldehyde reductive amination | 1.2.1 |
| 1 | 0.9007 | 0.8382 | 0.8683 | Eschweiler-Clarke methylation | 1.2.4 |
| 2 | 0.7287 | 0.7866 | 0.7565 | Ketone reductive amination | 1.2.5 |
| 3 | 0.6345 | 0.6095 | 0.6217 | Bromo N-arylation | 1.3.6 |
| 4 | 0.6837 | 0.6252 | 0.6531 | Chloro N-arylation | 1.3.7 |
| 5 | 0.7637 | 0.6424 | 0.6978 | Fluoro N-arylation | 1.3.8 |
| 6 | 0.5756 | 0.5717 | 0.5736 | Bromo N-alkylation | 1.6.2 |
| 7 | 0.574 | 0.3665 | 0.4474 | Chloro N-alkylation | 1.6.4 |
| 8 | 0.5724 | 0.6 | 0.5859 | Iodo N-alkylation | 1.6.8 |
| 9 | 0.8974 | 0.8714 | 0.8842 | Hydroxy to methoxy | 1.7.4 |
| 10 | 0.7368 | 0.7106 | 0.7235 | Methyl esterification | 1.7.6 |
| 11 | 0.5789 | 0.5166 | 0.546 | Mitsunobu aryl ether synthesis | 1.7.7 |
| 12 | 0.5429 | 0.4848 | 0.5122 | Williamson ether synthesis | 1.7.9 |
| 13 | 0.7976 | 0.9029 | 0.847 | Thioether synthesis | 1.8.5 |
| 14 | 0.9018 | 0.9352 | 0.9182 | Bromination | 10.1.1 |
| 15 | 0.9492 | 0.9554 | 0.9523 | Chlorination | 10.1.2 |
| 16 | 0.9547 | 0.9067 | 0.9301 | Wohl-Ziegler bromination | 10.1.5 |
| 17 | 0.9688 | 0.9748 | 0.9718 | Nitration | 10.2.1 |
| 18 | 0.6904 | 0.6856 | 0.688 | Methylation | 10.4.2 |
| 19 | 0.5394 | 0.5991 | 0.5677 | Amide Schotten-Baumann | 2.1.1 |
| 20 | 0.6417 | 0.557 | 0.5963 | Carboxylic acid + amine reaction | 2.1.2 |
| 21 | 0.9405 | 0.8614 | 0.8992 | N-acetylation | 2.1.7 |
| 22 | 0.8335 | 0.8039 | 0.8184 | Sulfonamide Schotten-Baumann | 2.2.3 |
| 23 | 0.843 | 0.9169 | 0.8784 | Isocyanate + amine reaction | 2.3.1 |
| 24 | 0.647 | 0.8422 | 0.7318 | Ester Schotten-Baumann | 2.6.1 |
| 25 | 0.659 | 0.6864 | 0.6724 | Fischer-Speier esterification | 2.6.3 |
| 26 | 0.7827 | 0.9351 | 0.8521 | Sulfonic ester Schotten-Baumann | 2.7.2 |
| 27 | 0.5771 | 0.4723 | 0.5195 | Bromo Suzuki coupling | 3.1.1 |
| 28 | 0.5179 | 0.5458 | 0.5315 | Bromo Suzuki-type coupling | 3.1.5 |
| 29 | 0.7774 | 0.7484 | 0.7627 | Chloro Suzuki-type coupling | 3.1.6 |
| 30 | 0.8246 | 0.9323 | 0.8751 | Sonogashira coupling | 3.3.1 |
| 31 | 0.6285 | 0.6217 | 0.6251 | Stille reaction | 3.4.1 |
| 32 | 0.9381 | 0.9811 | 0.9592 | N-Boc protection | 5.1.1 |
| 33 | 0.9344 | 0.9623 | 0.9482 | N-Boc deprotection | 6.1.1 |
| 34 | 0.9304 | 0.9218 | 0.926 | N-Cbz deprotection | 6.1.3 |
| 35 | 0.9848 | 0.9676 | 0.9761 | N-Bn deprotection | 6.1.5 |
| 36 | 0.8134 | 0.7943 | 0.8037 | CO2H-Et deprotection | 6.2.1 |
| 37 | 0.7904 | 0.7835 | 0.7869 | CO2H-Me deprotection | 6.2.2 |
| 38 | 0.9573 | 0.9514 | 0.9543 | CO2H-tBu deprotection | 6.2.3 |
| 39 | 0.9173 | 0.9494 | 0.9331 | O-Bn deprotection | 6.3.1 |
| 40 | 0.9418 | 0.9625 | 0.9521 | Methoxy to hydroxy | 6.3.7 |
| 41 | 0.985 | 0.985 | 0.985 | Nitro to amino | 7.1.1 |
| 42 | 0.9595 | 0.9922 | 0.9756 | Amide to amine reduction | 7.2.1 |
| 43 | 0.9975 | 0.9925 | 0.995 | Nitrile reduction | 7.3.1 |
| 44 | 0.9898 | 0.9987 | 0.9943 | Carboxylic acid to alcohol reduction | 7.9.2 |
| 45 | 0.9887 | 0.9825 | 0.9856 | Alcohol to aldehyde oxidation | 8.1.4 |
| 46 | 0.977 | 0.9782 | 0.9776 | Alcohol to ketone oxidation | 8.1.5 |
| 47 | 0.9912 | 0.973 | 0.982 | Sulfanyl to sulfinyl | 8.2.1 |
| 48 | 0.9697 | 0.9796 | 0.9747 | Hydroxy to chloro | 9.1.6 |
| 49 | 0.9975 | 0.99 | 0.9938 | Carboxylic acid to acid chloride | 9.3.1 |

**Table S7.** The *rxnfp* (10k)'s performance on the Schneider 50k dataset.

| **Index** | **Recall** | **Precision** | **F1-Score** | **Class Name** | **Class** |
| --- | --- | --- | --- | --- | --- |
| 0 | 0.7886 | 0.8489 | 0.8177 | Aldehyde reductive amination | 1.2.1 |
| 1 | 0.9882 | 0.9692 | 0.9786 | Eschweiler-Clarke methylation | 1.2.4 |
| 2 | 0.8643 | 0.9266 | 0.8944 | Ketone reductive amination | 1.2.5 |
| 3 | 0.8111 | 0.7004 | 0.7517 | Bromo N-arylation | 1.3.6 |
| 4 | 0.7466 | 0.7715 | 0.7589 | Chloro N-arylation | 1.3.7 |
| 5 | 0.8025 | 0.8243 | 0.8132 | Fluoro N-arylation | 1.3.8 |
| 6 | 0.701 | 0.7096 | 0.7053 | Bromo N-alkylation | 1.6.2 |
| 7 | 0.7558 | 0.5717 | 0.651 | Chloro N-alkylation | 1.6.4 |
| 8 | 0.6941 | 0.7081 | 0.701 | Iodo N-alkylation | 1.6.8 |
| 9 | 0.9896 | 0.9609 | 0.975 | Hydroxy to methoxy | 1.7.4 |
| 10 | 0.8309 | 0.7143 | 0.7682 | Methyl esterification | 1.7.6 |
| 11 | 0.6622 | 0.7392 | 0.6986 | Mitsunobu aryl ether synthesis | 1.7.7 |
| 12 | 0.7054 | 0.57 | 0.6305 | Williamson ether synthesis | 1.7.9 |
| 13 | 0.9462 | 0.9509 | 0.9485 | Thioether synthesis | 1.8.5 |
| 14 | 0.9452 | 0.9648 | 0.9549 | Bromination | 10.1.1 |
| 15 | 0.987 | 0.9781 | 0.9825 | Chlorination | 10.1.2 |
| 16 | 0.9723 | 0.9496 | 0.9608 | Wohl-Ziegler bromination | 10.1.5 |
| 17 | 0.99 | 0.9826 | 0.9863 | Nitration | 10.2.1 |
| 18 | 0.8884 | 0.7845 | 0.8332 | Methylation | 10.4.2 |
| 19 | 0.7493 | 0.7975 | 0.7727 | Amide Schotten-Baumann | 2.1.1 |
| 20 | 0.7479 | 0.6233 | 0.6799 | Carboxylic acid + amine reaction | 2.1.2 |
| 21 | 0.9905 | 0.8853 | 0.9349 | N-acetylation | 2.1.7 |
| 22 | 0.914 | 0.9653 | 0.939 | Sulfonamide Schotten-Baumann | 2.2.3 |
| 23 | 0.912 | 0.9734 | 0.9417 | Isocyanate + amine reaction | 2.3.1 |
| 24 | 0.8556 | 0.9217 | 0.8874 | Ester Schotten-Baumann | 2.6.1 |
| 25 | 0.6436 | 0.7577 | 0.696 | Fischer-Speier esterification | 2.6.3 |
| 26 | 0.8279 | 0.9801 | 0.8976 | Sulfonic ester Schotten-Baumann | 2.7.2 |
| 27 | 0.5514 | 0.409 | 0.4697 | Bromo Suzuki coupling | 3.1.1 |
| 28 | 0.4787 | 0.5746 | 0.5223 | Bromo Suzuki-type coupling | 3.1.5 |
| 29 | 0.7613 | 0.7108 | 0.7352 | Chloro Suzuki-type coupling | 3.1.6 |
| 30 | 0.967 | 0.969 | 0.968 | Sonogashira coupling | 3.3.1 |
| 31 | 0.6834 | 0.7663 | 0.7225 | Stille reaction | 3.4.1 |
| 32 | 0.9613 | 0.979 | 0.9701 | N-Boc protection | 5.1.1 |
| 33 | 0.995 | 0.9962 | 0.9956 | N-Boc deprotection | 6.1.1 |
| 34 | 0.992 | 0.9987 | 0.9953 | N-Cbz deprotection | 6.1.3 |
| 35 | 0.9987 | 0.9987 | 0.9987 | N-Bn deprotection | 6.1.5 |
| 36 | 0.9975 | 0.9962 | 0.9968 | CO2H-Et deprotection | 6.2.1 |
| 37 | 0.9987 | 0.99 | 0.9943 | CO2H-Me deprotection | 6.2.2 |
| 38 | 0.9937 | 1 | 0.9969 | CO2H-tBu deprotection | 6.2.3 |
| 39 | 0.99 | 0.9962 | 0.9931 | O-Bn deprotection | 6.3.1 |
| 40 | 0.9949 | 0.9962 | 0.9956 | Methoxy to hydroxy | 6.3.7 |
| 41 | 0.9937 | 0.9913 | 0.9925 | Nitro to amino | 7.1.1 |
| 42 | 0.9937 | 0.9949 | 0.9943 | Amide to amine reduction | 7.2.1 |
| 43 | 1 | 0.9987 | 0.9994 | Nitrile reduction | 7.3.1 |
| 44 | 0.9962 | 0.9949 | 0.9956 | Carboxylic acid to alcohol reduction | 7.9.2 |
| 45 | 1 | 0.9987 | 0.9994 | Alcohol to aldehyde oxidation | 8.1.4 |
| 46 | 0.9962 | 0.9974 | 0.9968 | Alcohol to ketone oxidation | 8.1.5 |
| 47 | 0.9988 | 0.9975 | 0.9981 | Sulfanyl to sulfinyl | 8.2.1 |
| 48 | 0.9887 | 0.9987 | 0.9937 | Hydroxy to chloro | 9.1.6 |
| 49 | 0.9987 | 0.9938 | 0.9962 | Carboxylic acid to acid chloride | 9.3.1 |

**Table S8.** Candidates for experimental verification.

| Candidate | Uniport ID |
| --- | --- |
| ZD1 | A0A9N9YUV3 |
| ZD2 | A0A1L7XUA8 |
| ZD3 | A0A1V8T0W6 |
| ZD4 | A0AA38Y7K6 |
| ZD5 | A0A0D2H1W2 |
| ZD6 | A0A1V1SRR3 |
| ZD7 | A0A8K0STV1 |
| ZD8 | A0A9P9QD81 |
| ZD9 | A0A2J6S3X0 |
| ZD10 | A0A370TT42 |

**Table S9.** Comparison of CACLENS with existing models in terms of capabilities

| **Model name** | **Reaction classification** | **EC number prediction** | **Reaction feasibility prediction** | **Experimental validation** | **Training time** | **GPU** | **Source** |
| --- | --- | --- | --- | --- | --- | --- | --- |
| ESP ^7^ | × | × | √ | × | Unknown | 6×NVIDIA DGX A100 (40GB) | *Nature Communications, 2023* |
| CLEAN ^8^ | × | √ | × | √ | Unknown | Unknown | *Science， 2023* |
| rxnfp ^4^ | √ | × | × | × | Unknown | Unknown | *Nature Machine Intelligence, 2021* |
| BEC-Pred ^9^ | × | √ | × | × | Unknown | Unknown | *Journal of Cheminformatics,2024* |
| PU-EPP ^10^ | × | × | √ | √ | 14 days | 5×NVIDIA Tesla V100 GPUs | *ACS Catalysis, 2024* |
| CAPLA ^11^ | × | × | √ (binding affinity) | × | Unknown | GTX1080Ti GPU. | *Bioinformatics, 2023* |
| Enzymatic Transformer ^12^ | × | × | √  (output product SMILES) | × | Unknown | Unknown | *Chemical Science, 2021* |
| FusionESP ^13^ | × | × | √ | × | Unknown | Tesla T4 GPU | *Journal of Chemical Information and Modeling, 2025* |
| MTL FP-GNN ^14^ | × | × | √ | × | Unknown | Unknown | *ChemcalResearch in Toxicology, 2024* |
| CPI-IGAE ^15^ | × | × | √  (binding affinity) | × | Unknown | Unknown | *Briefings in Bioinformatics, 2022* |
| CACLENS | √ | √ | √ | √ | 1.5days | NVIDIA DGX A100 (40GB) | *-* |

**Reference**

1 Jain, S. M. in *Introduction to transformers for NLP: With the hugging face library and models to solve problems* 51-67 (Springer, 2022).

2 Bepler, T. & Berger, B. Learning the protein language: Evolution, structure, and function. *Cell systems* **12**, 654-669. e653 (2021).

3 Wei, J.-M., Yuan, X.-J., Hu, Q.-H. & Wang, S.-Q. A novel measure for evaluating classifiers. *Expert Systems with Applications* **37**, 3799-3809 (2010).

4 Schwaller, P. *et al.* Mapping the space of chemical reactions using attention-based neural networks. *Nature Machine Intelligence* **3**, 144-152 (2021). <https://doi.org/10.1038/s42256-020-00284-w>

5 Matthews, B. W. Comparison of the predicted and observed secondary structure of T4 phage lysozyme. *Biochimica et Biophysica Acta (BBA)-Protein Structure* **405**, 442-451 (1975).

6 Gorodkin, J. Comparing two K-category assignments by a K-category correlation coefficient. *Computational biology and chemistry* **28**, 367-374 (2004).

7 Kroll, A., Ranjan, S., Engqvist, M. K. M. & Lercher, M. J. A general model to predict small molecule substrates of enzymes based on machine and deep learning. *Nature Communications* **14** (2023). <https://doi.org/10.1038/s41467-023-38347-2>

8 Yu, T. *et al.* Enzyme function prediction using contrastive learning. *Science* **379**, 1358-+ (2023). <https://doi.org/10.1126/science.adf2465>

9 Qian, W. *et al.* A general model for predicting enzyme functions based on enzymatic reactions. *Journal of Cheminformatics* **16**, 38 (2024).

10 Zhang, D. *et al.* Discovery of toxin-degrading enzymes with positive unlabeled deep learning. *Acs Catalysis* **14**, 3336-3348 (2024).

11 Jin, Z. *et al.* CAPLA: improved prediction of protein–ligand binding affinity by a deep learning approach based on a cross-attention mechanism. *Bioinformatics* **39**, btad049 (2023).

12 Kreutter, D., Schwaller, P. & Reymond, J.-L. Predicting enzymatic reactions with a molecular transformer. *Chemical science* **12**, 8648-8659 (2021).

13 Du, Z., Fu, W., Guo, X., Caragea, D. & Li, Y. FusionESP: Improved Enzyme–Substrate Pair Prediction by Fusing Protein and Chemical Knowledge. *Journal of Chemical Information and Modeling* (2025).

14 Fang, J. *et al.* Prediction of cytochrome p450 substrates using the explainable multitask deep learning models. *Chemical Research in Toxicology* **37**, 1535-1548 (2024).

15 Wan, X. *et al.* An inductive graph neural network model for compound–protein interaction prediction based on a homogeneous graph. *Briefings in Bioinformatics* **23**, bbac073 (2022).
